# Supplementary material for: Molecular mechanism targeting condensin for chromosome condensation
Source: EMBO J. 2024 Dec 17;44(3):705–35. doi: 10.1038/s44318-024-00336-6 (PMC11791182; doi:10.1038/s44318-024-00336-6)
Supplement: Supplementary file 1 — Appendix [file 44318_2024_336_MOESM1_ESM.pdf]

Molecular mechanism targeting condensin for chromosome condensation

Menglu Wang, Daniel Robertson, Juan Zou, Christos Spanos, Juri Rappsilber, and Adele L. Marston

Appendix

Table of Contents

p. 2 - Appendix Figure S1. Mutation of Sgo1 Conserved Region 1 (CR1) disrupts condensin recruitment to an ectopic site.
p. 3 - Appendix Figure S2. PP2A and cohesin association with Sgo1 is not affected by disruption of the CR1-Ycg1 interface.
p. 4 - Appendix Figure S3. Hi-C heat map of borders.
p. 5 - Appendix Figure S4. Hi-C heat map along chromosome arms.
p. 6 - Appendix Figure S5. Hi-C heat map of ChrXII.

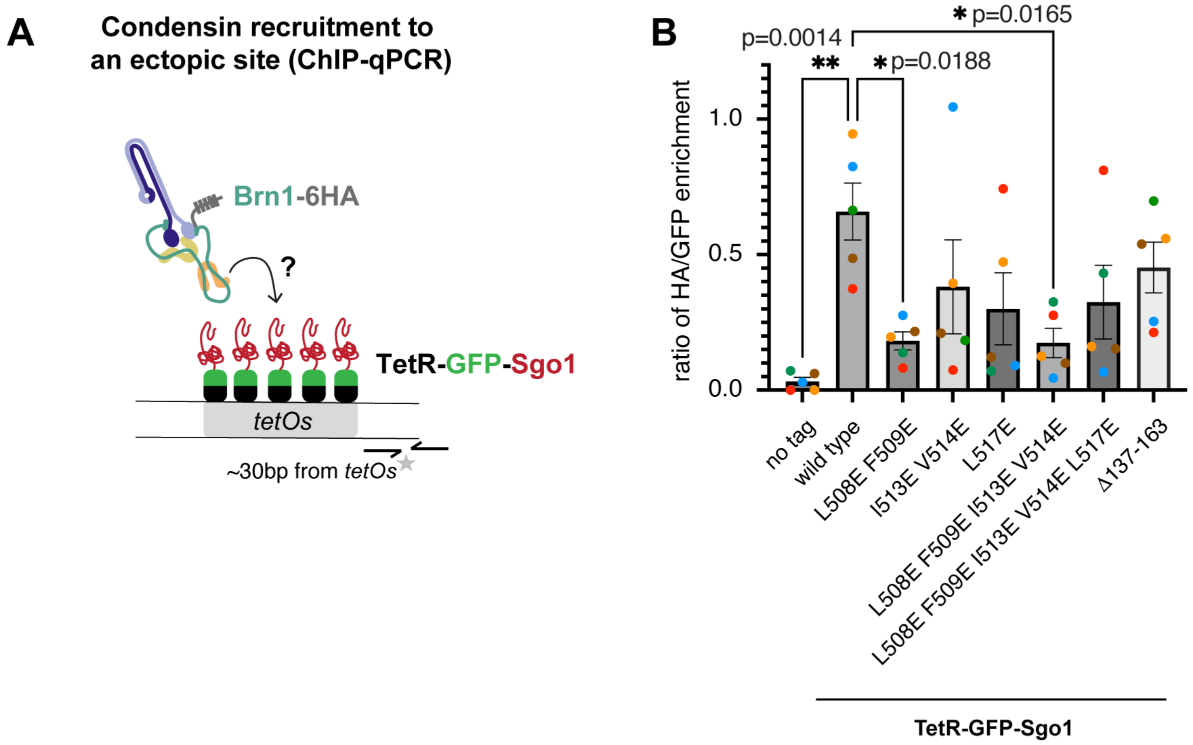

Appendix Figure S1. Mutation of Sgo1 Conserved Region 1 (CR1) disrupts condensin recruitment to an ectopic site.

A, Schematic diagram showing tethered Sgo1 at an ectopic site recruits condensin, grey asterisk indicates the position of primers used for qPCR analysis, ~30 bp distant from the tethering site.

B, ChIP-qPCR measuring the levels of Brn1-6HA recruited to the tethered wild type and mutant Sgo1 variants. Strains were arrested in mitosis by treatment with nocodazole and benomyl for 2 hours. The ratios of Brn1 and Sgo1 enrichment (anti-HA/anti-GFP ChIP-qPCR values were determined and the mean of four experimental repeats is shown with error bars representing standard error. *$p<0.0332$; **$p<0.0021$, one-way ordinary ANOVA with Dunnetts correction, only comparisons significantly different from wild type are indicated. Strains used: no tag for anti-HA ChIP-qPCR (AM9655), no tag for anti-GFP ChIP-qPCR (AM20092), wild type (AM9847), L508E F509E (AM32194), I513E V514E (AM32195), L517E (AM32196), L508E F509E I513E V514E (AM32197), L508E F509E I513E V514E L517E (AM32198), Δ137-163 (AM32199).

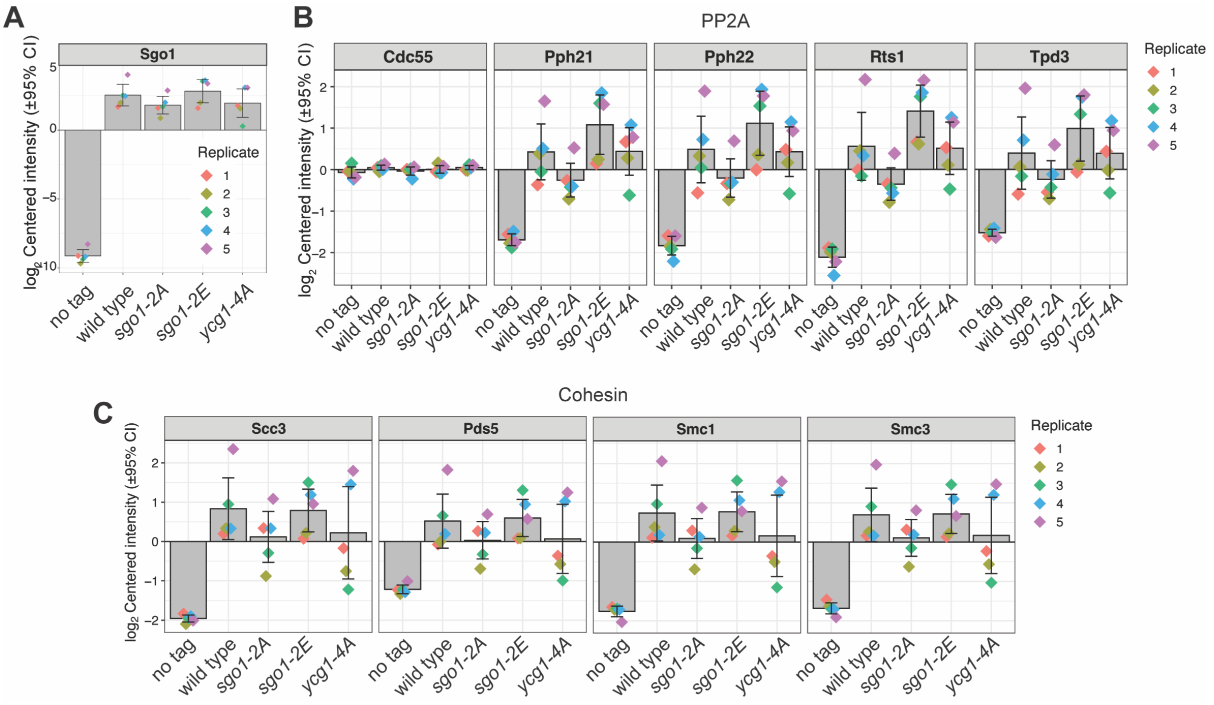

Appendix Figure S2. PP2A and cohesin association with Sgo1 is not affected by disruption of the CR1-Ycg1 interface.

Abundance of Sgo1 (**A**), PP2A (**B**), cohesin (**C**) in Sgo1-FLAG IP-MS. Plots show intensity values scaled to the mean of all conditions on a log_2 scale and therefore represent relative rather than absolute comparisons. Error bars represent the 95% confidence intervals. Data represents values from 5 biological replicates.

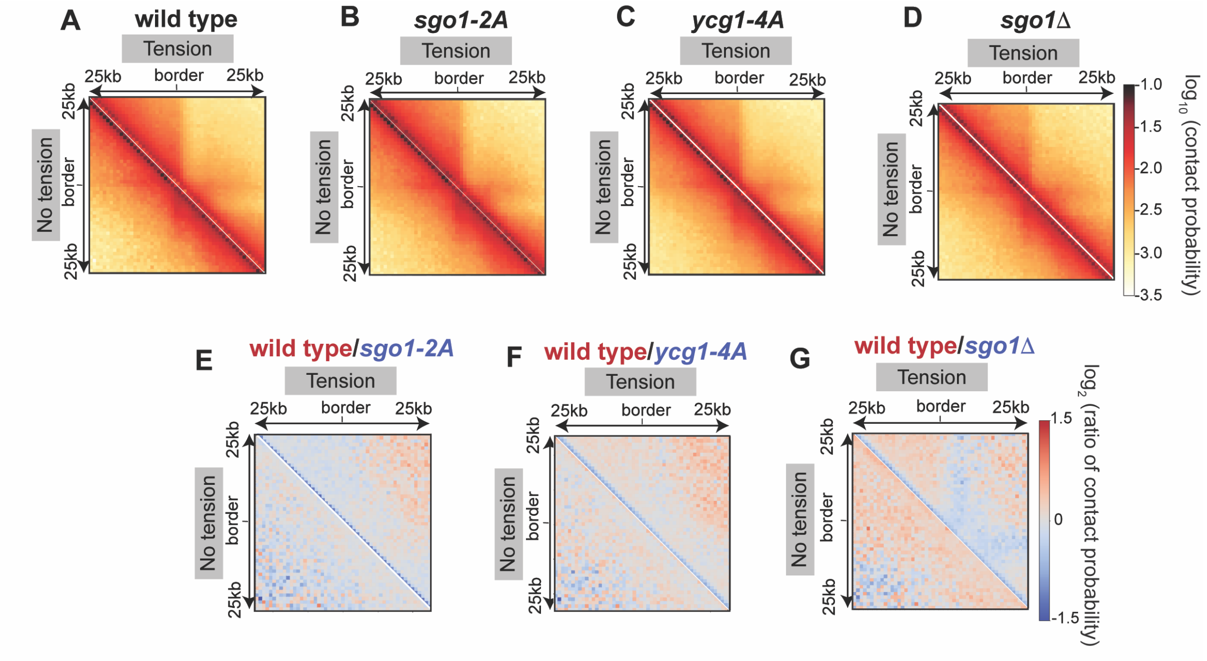

Appendix Figure S3. Hi-C heat map of borders.

A-G, Pile-ups (**A-D**, share the same scale bar on the right) and $\log_2$ ratio maps (**E-G**, share the same scale bar on the right) of *cis* contacts 25 kb surrounding all 32 borders for cells in the presence (upper right) or absence (lower left) of tension.

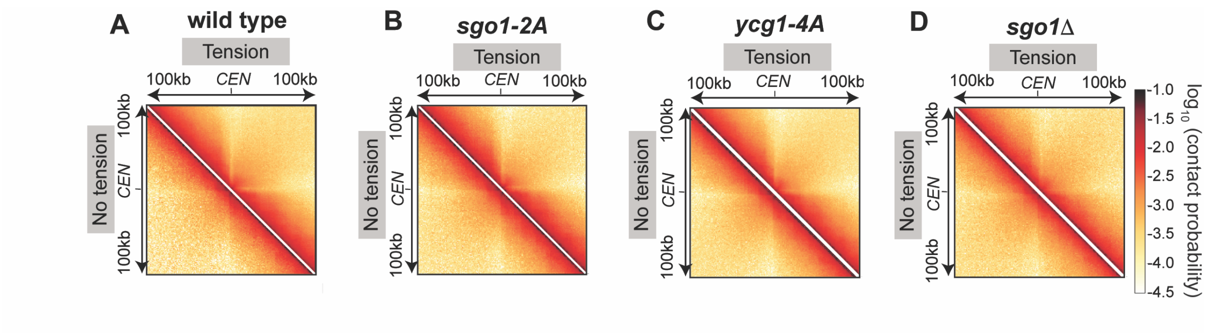

Appendix Figure S4. Hi-C heat map along chromosome arms.

A-D, Pile-ups (bin size 1kb) of *cis* contacts 100 kb surrounding all 16 centromeres for wild type, *sgo1-2A*, *ycg1-4A* and *sgo1Δ* in metaphase-arrested cells without spindle tension (lower left of the heatmap) or with tension (upper right). All Hi-C maps share the same scale bar on the right.

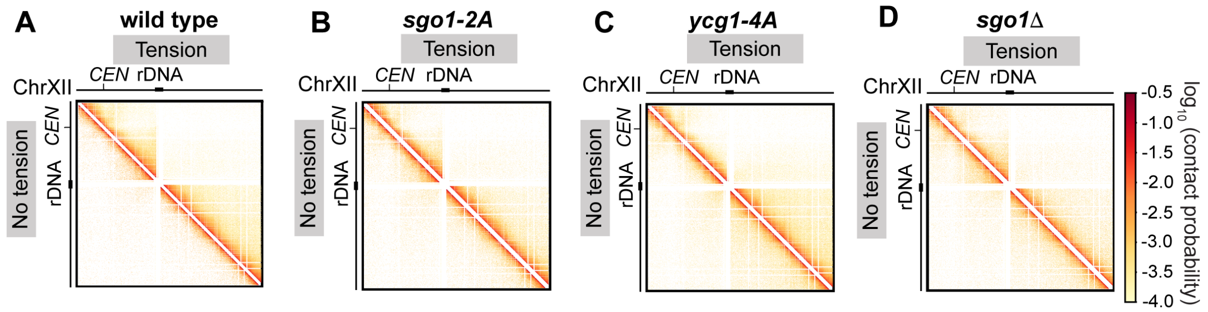

Appendix Figure S5. Hi-C heat map of ChrXII.

A-D, Hi-C maps of Chromosome XII in metaphase-arrested cells in the absence (lower left) and in the presence (upper right) of tension. All Hi-C maps share the same scale bar on the right.
