# Supplementary figures and images for: Molecular mechanism targeting condensin for chromosome condensation
Source: EMBO J. 2024 Dec 17;44(3):705–35. doi: 10.1038/s44318-024-00336-6 (PMC11791182; doi:10.1038/s44318-024-00336-6)

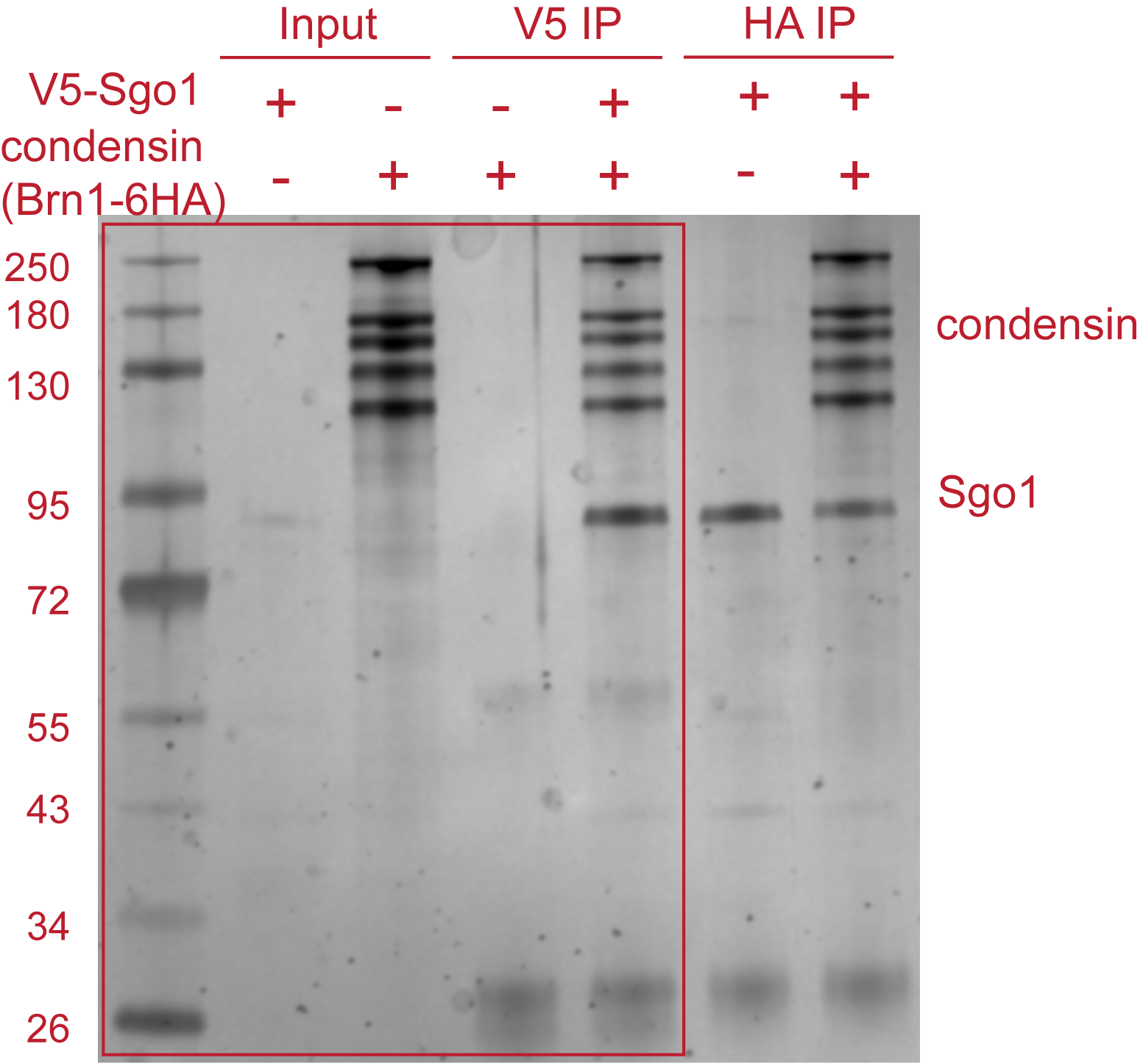

Supplement: Supplementary file 8 — Source data Fig. 1 [file 44318_2024_336_MOESM8_ESM.zip › SD Figure1/1B/1B silver stain.tif]

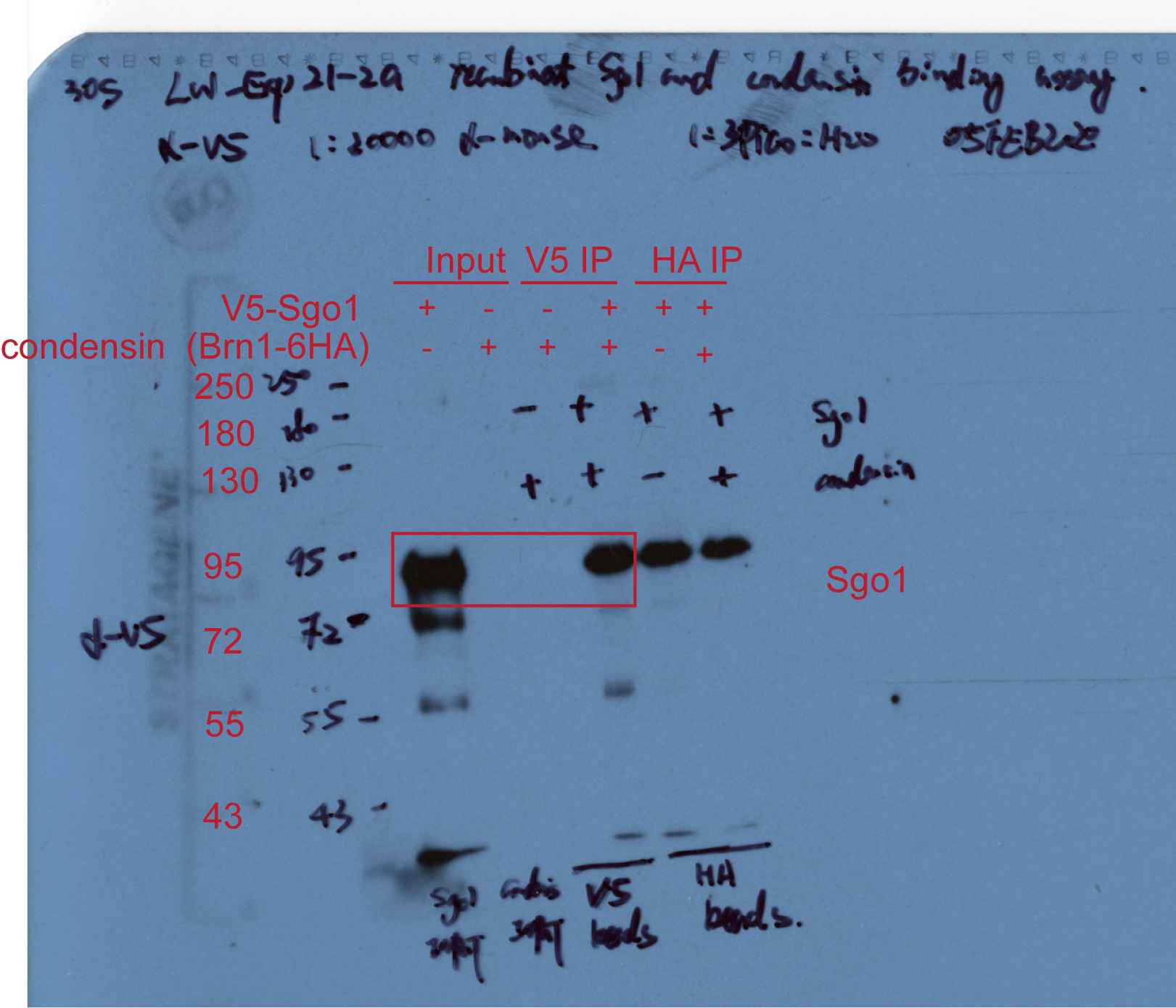

Supplement: Supplementary file 8 — Source data Fig. 1 [file 44318_2024_336_MOESM8_ESM.zip › SD Figure1/1C/1C western SGO1.tif]

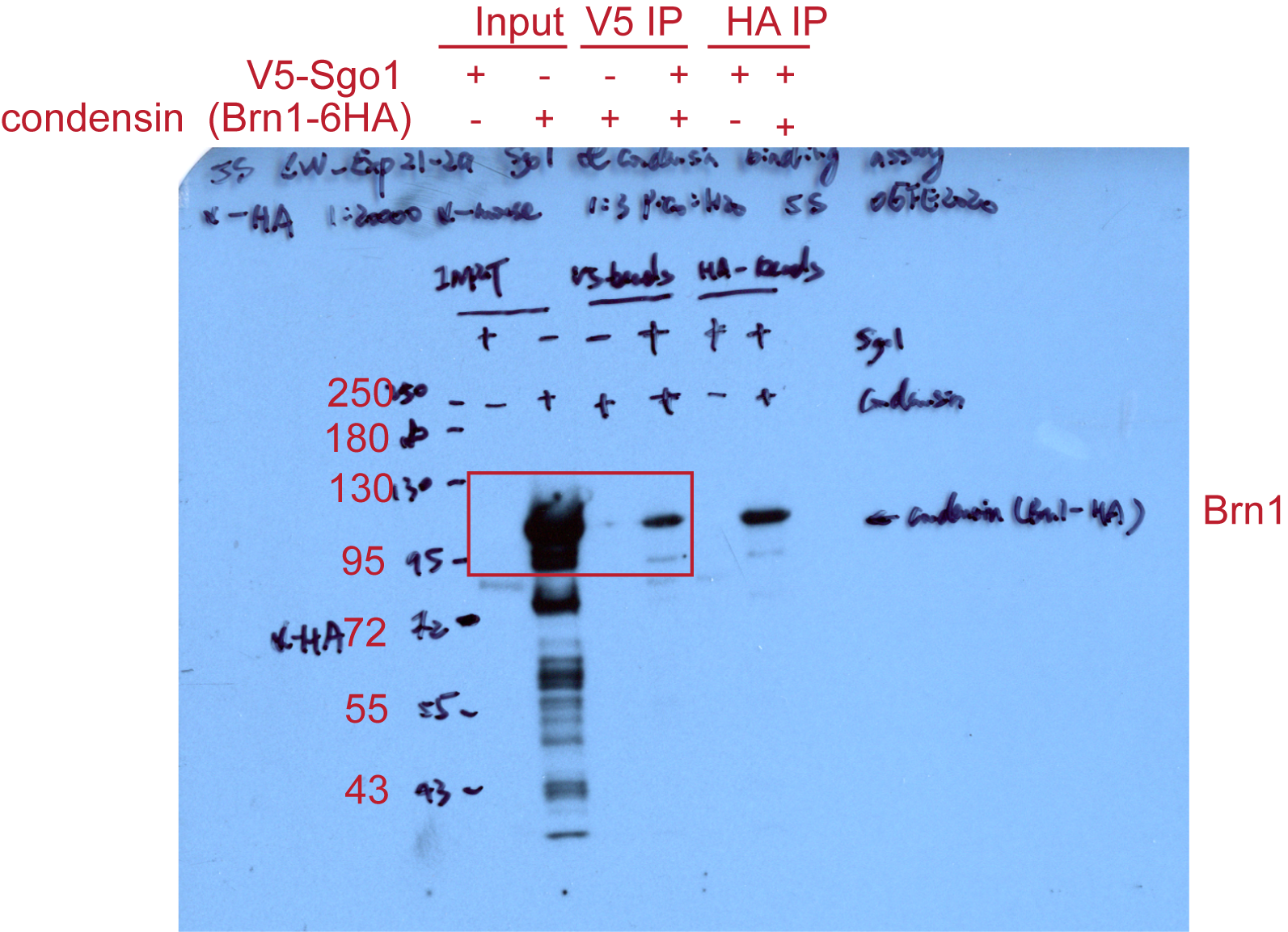

Supplement: Supplementary file 8 — Source data Fig. 1 [file 44318_2024_336_MOESM8_ESM.zip › SD Figure1/1C/1C western BRN1.tif]

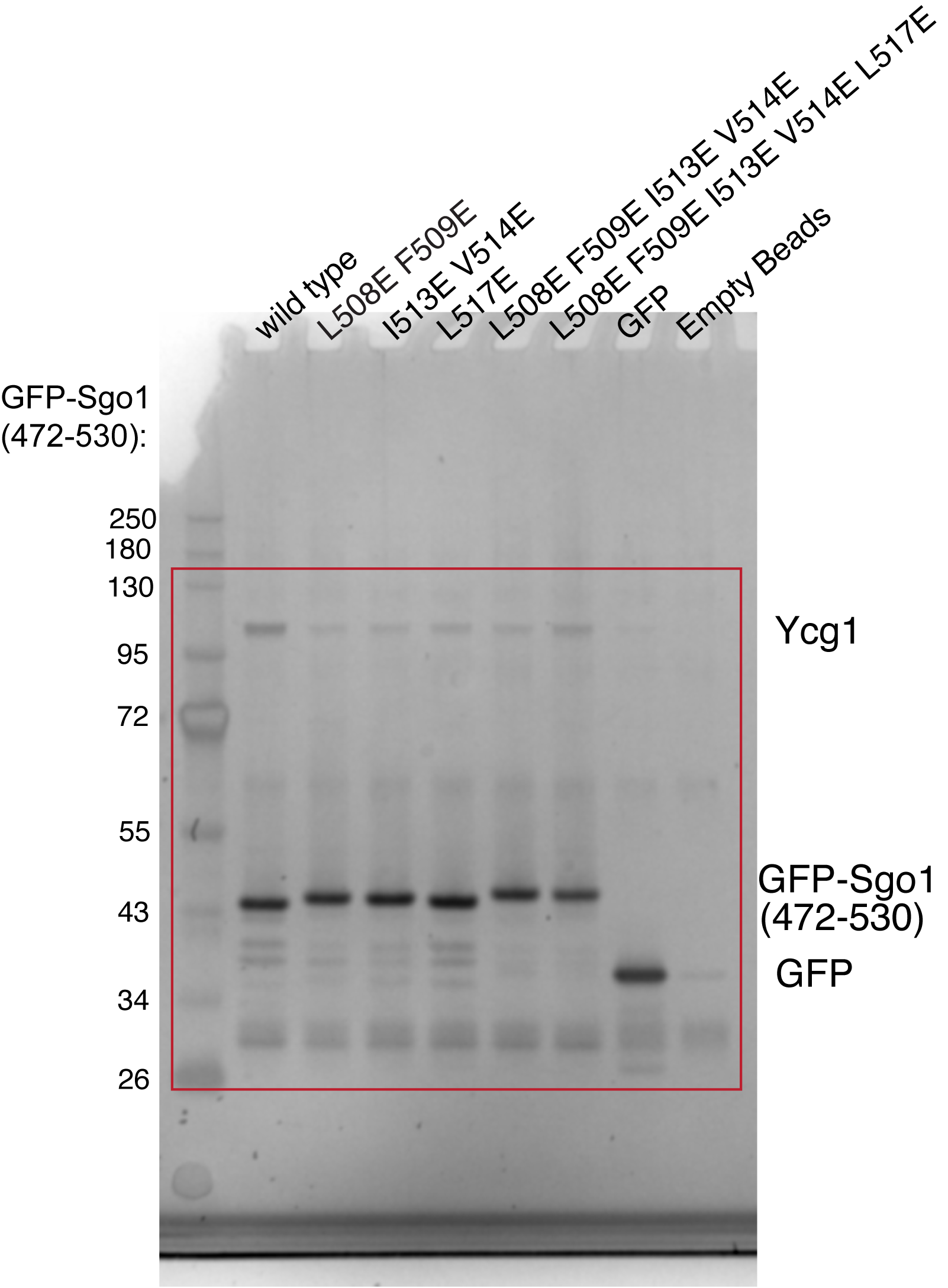

Supplement: Supplementary file 9 — Source data Fig. 2 [file 44318_2024_336_MOESM9_ESM.zip › SD Figure2/2G/2G silver stain.tif]

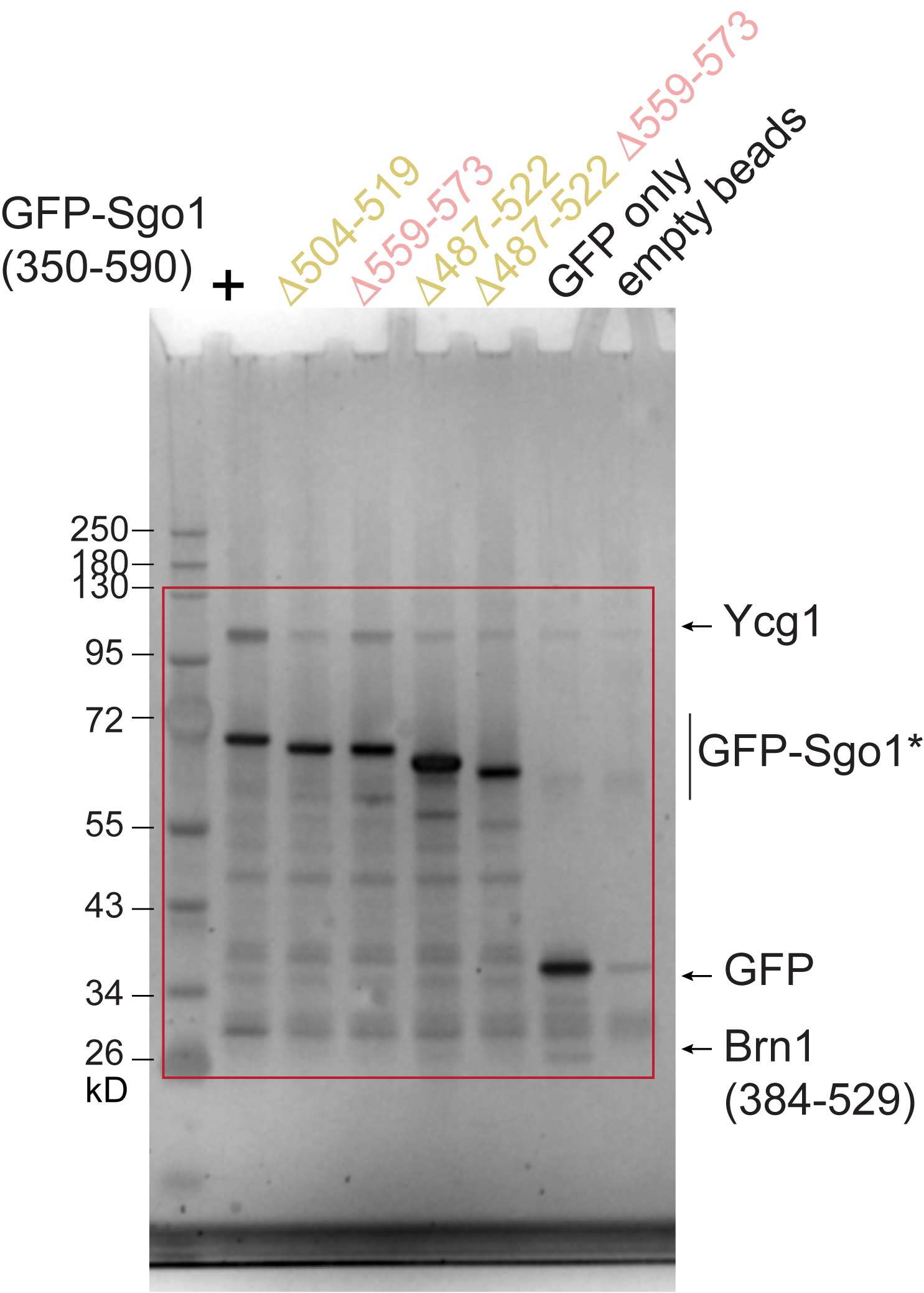

Supplement: Supplementary file 9 — Source data Fig. 2 [file 44318_2024_336_MOESM9_ESM.zip › SD Figure2/2D/2D silver stain.tif]

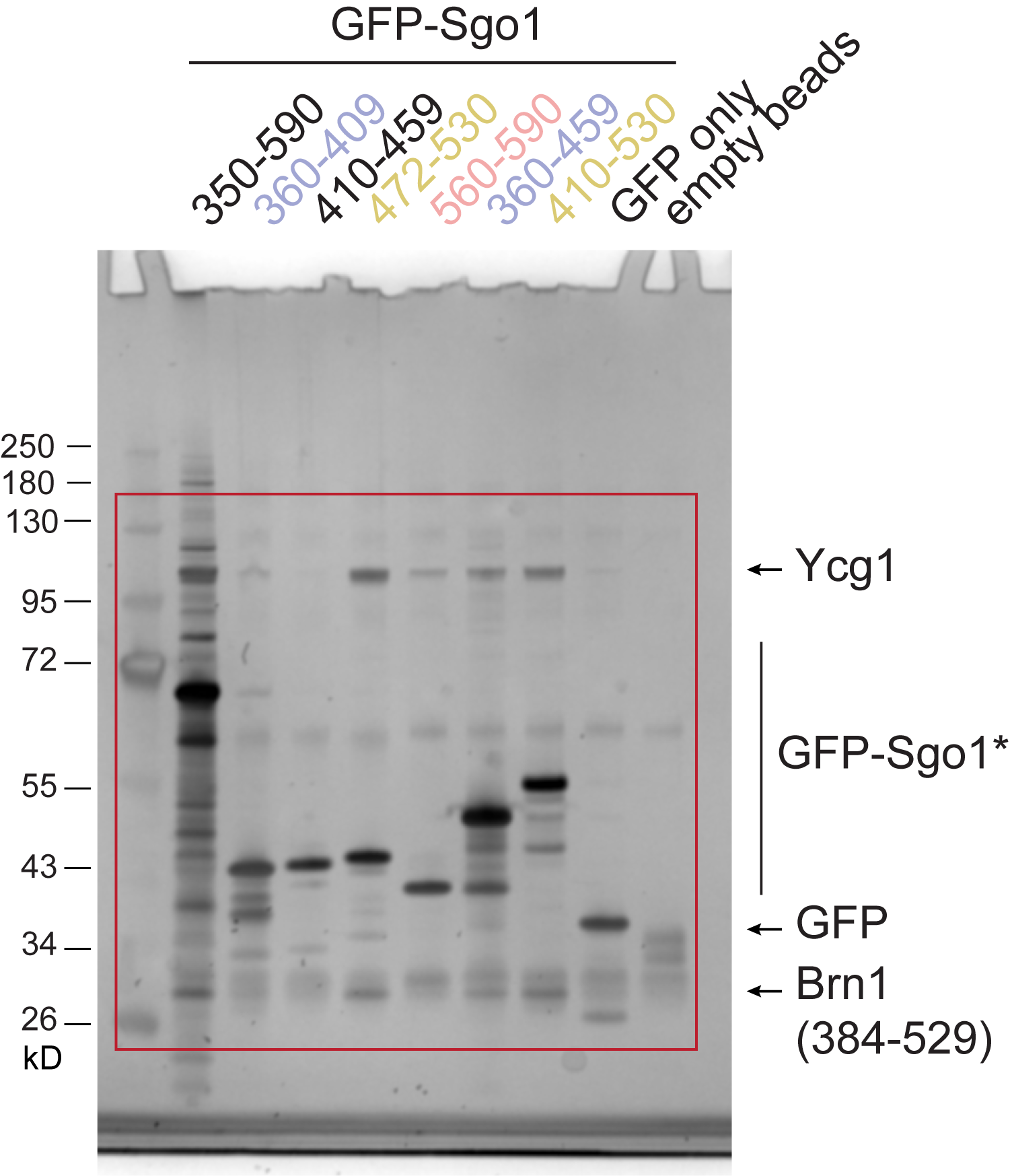

Supplement: Supplementary file 9 — Source data Fig. 2 [file 44318_2024_336_MOESM9_ESM.zip › SD Figure2/2B/2B silver stain.tif]

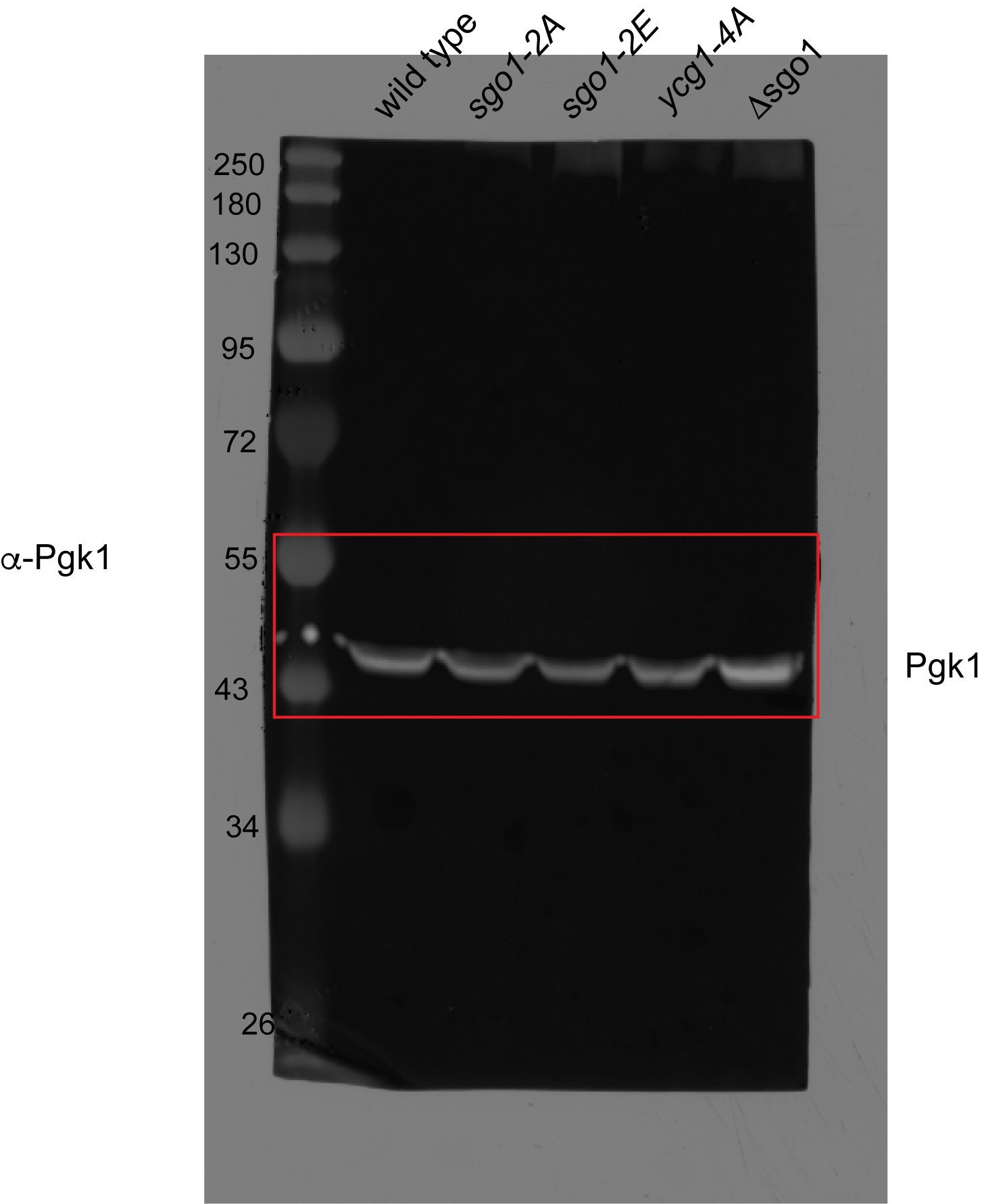

Supplement: Supplementary file 10 — Source data Fig. 5 [file 44318_2024_336_MOESM10_ESM.zip › SD Figure5/5B western Pgk1.tif]

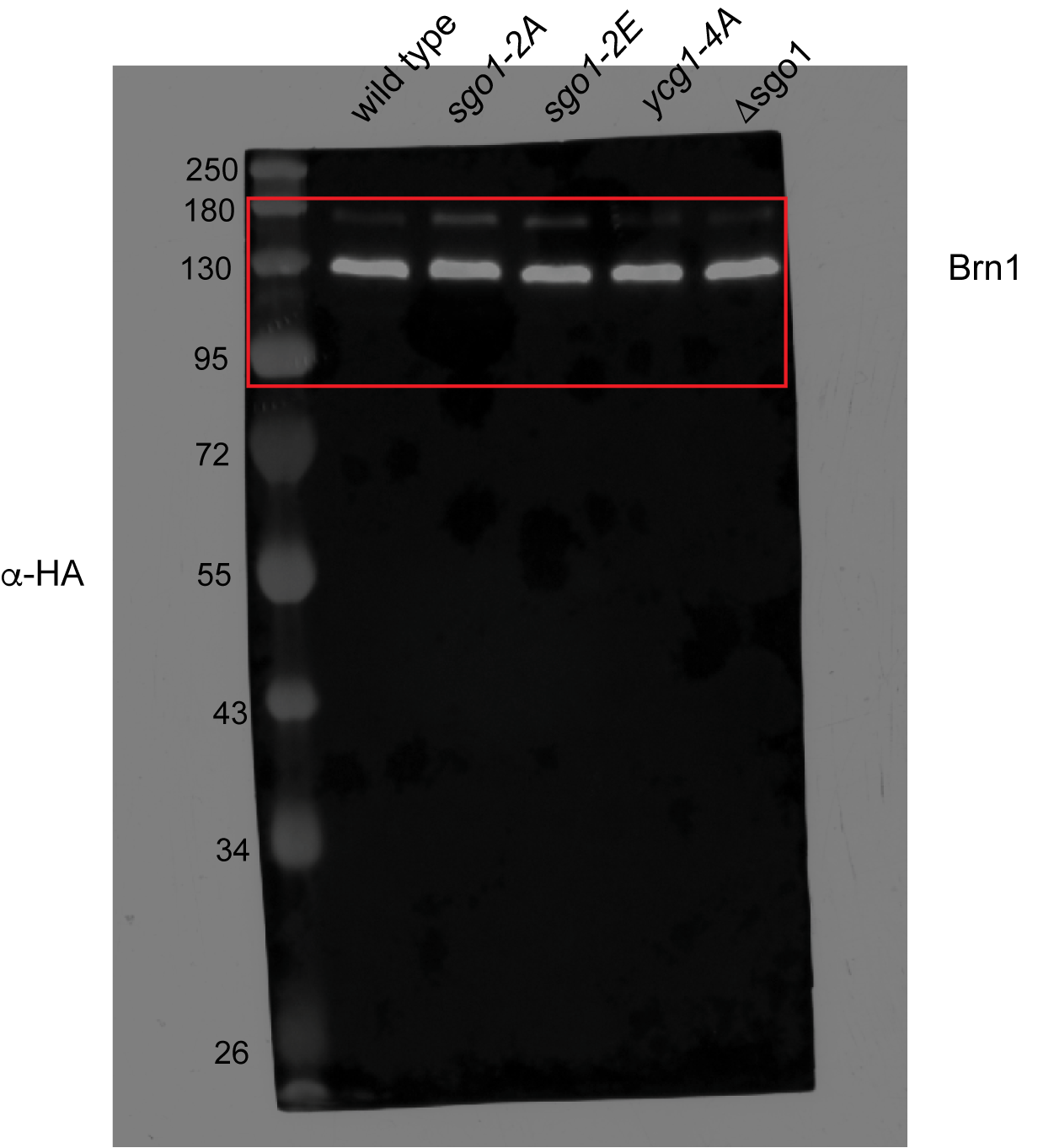

Supplement: Supplementary file 10 — Source data Fig. 5 [file 44318_2024_336_MOESM10_ESM.zip › SD Figure5/5B western Brn1-HA.tif]

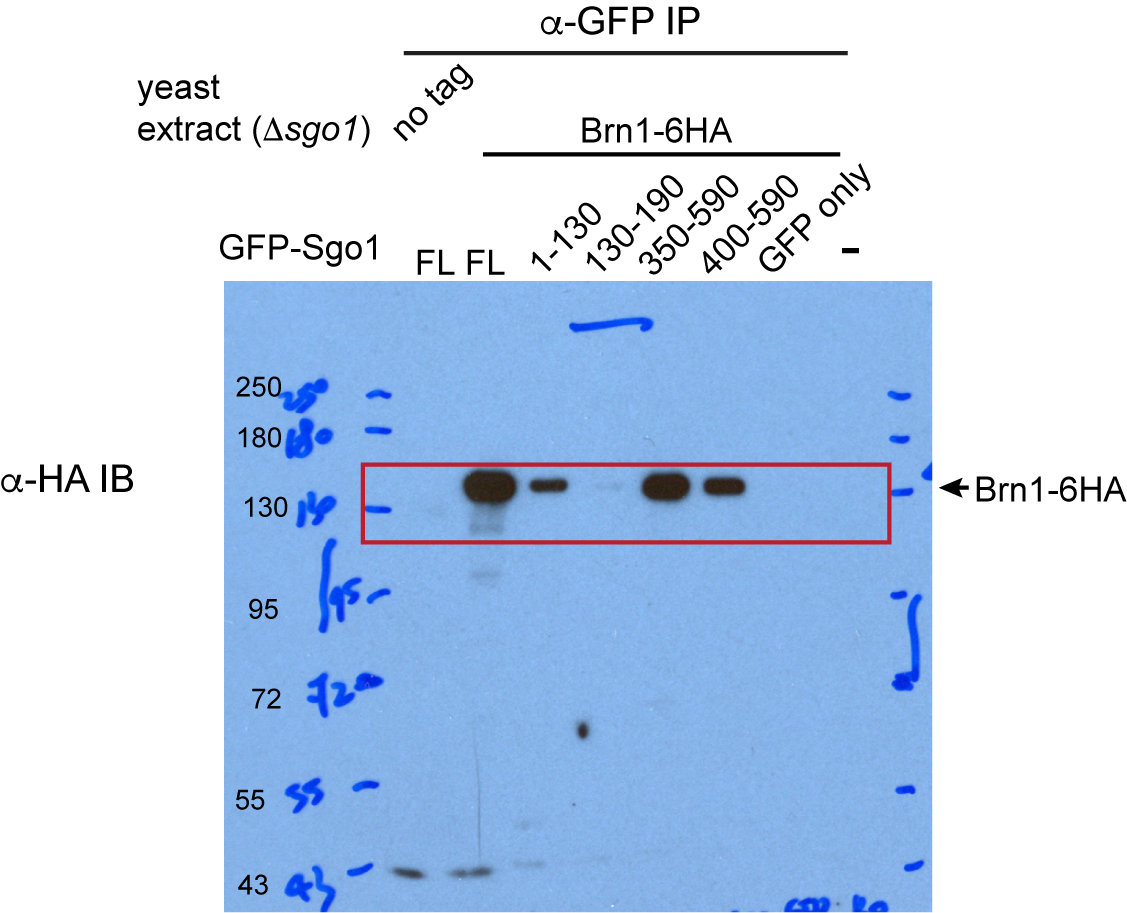

Supplement: Supplementary file 13 — EV Figure Source Data [file 44318_2024_336_MOESM13_ESM.zip › SD EV Figures/FigureEV1/EV1C western IP Brn1-HA.tif]

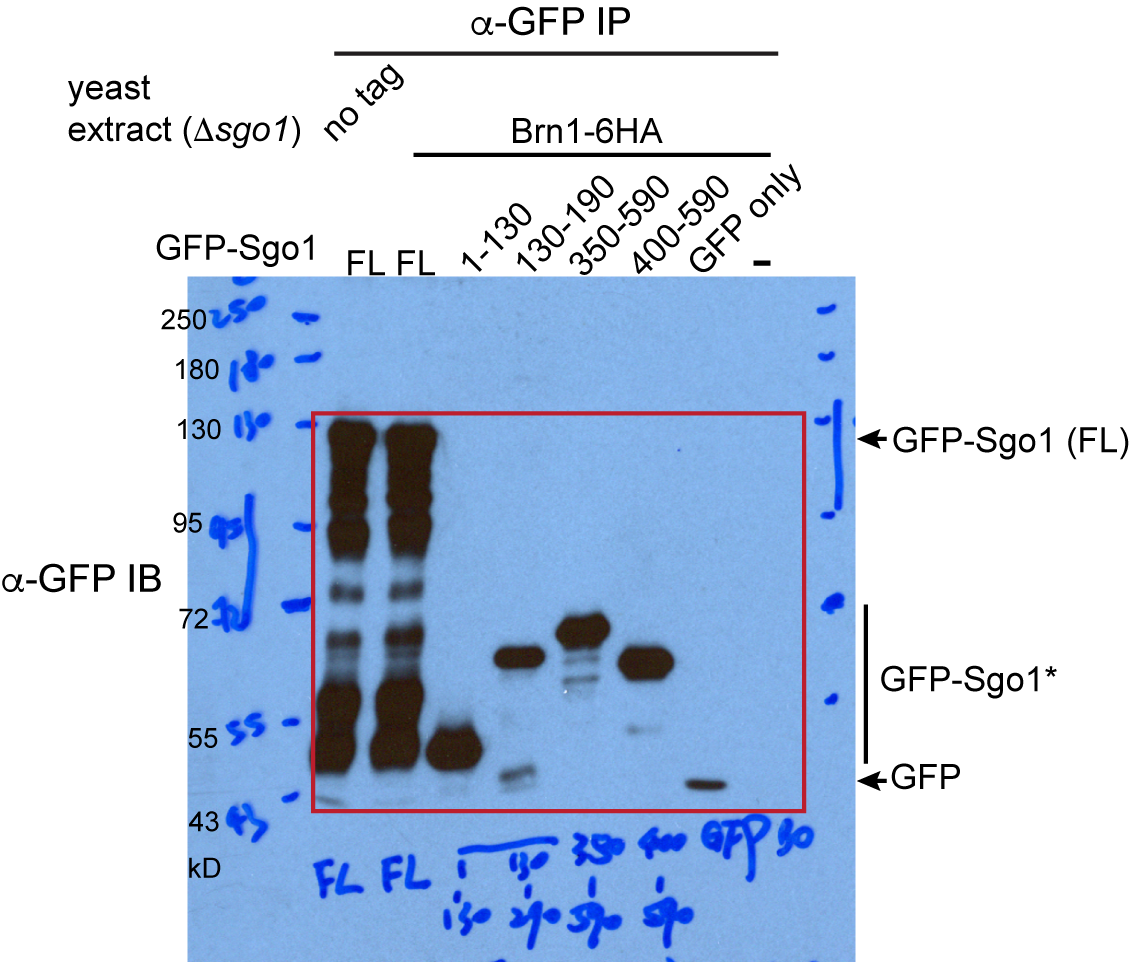

Supplement: Supplementary file 13 — EV Figure Source Data [file 44318_2024_336_MOESM13_ESM.zip › SD EV Figures/FigureEV1/EV1C western IP GFP-Sgo1.tif]

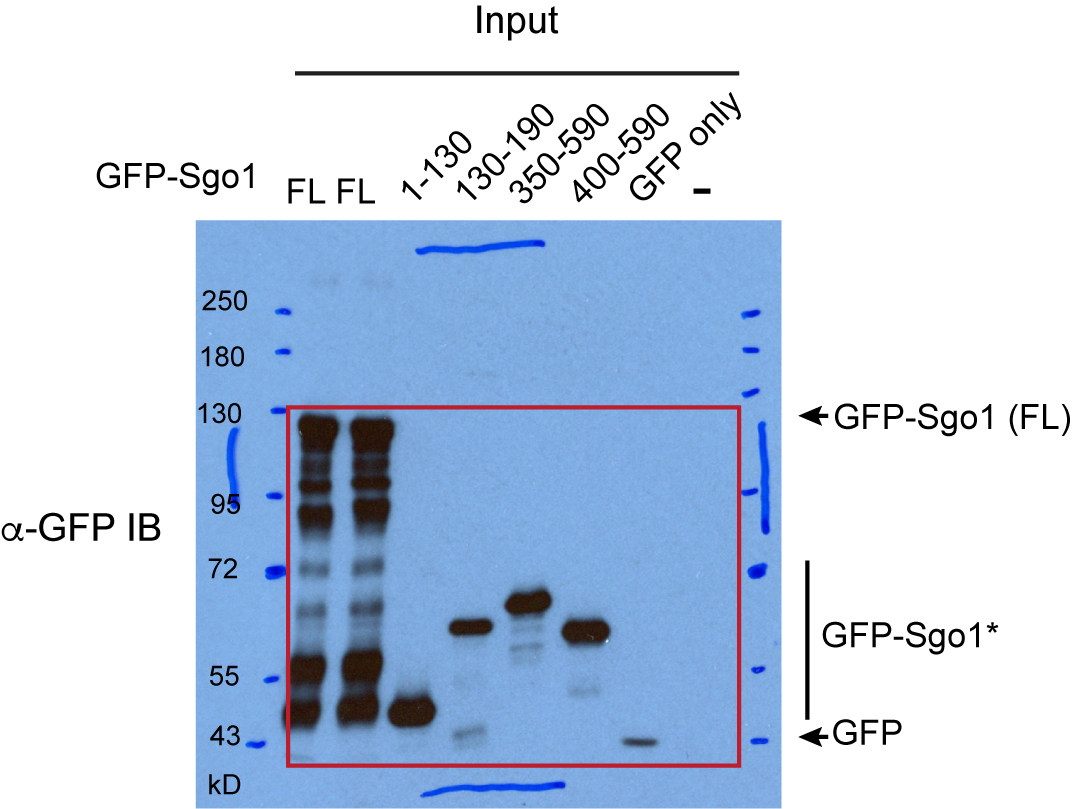

Supplement: Supplementary file 13 — EV Figure Source Data [file 44318_2024_336_MOESM13_ESM.zip › SD EV Figures/FigureEV1/EV1C western INPUT GFP-Sgo1.tif]

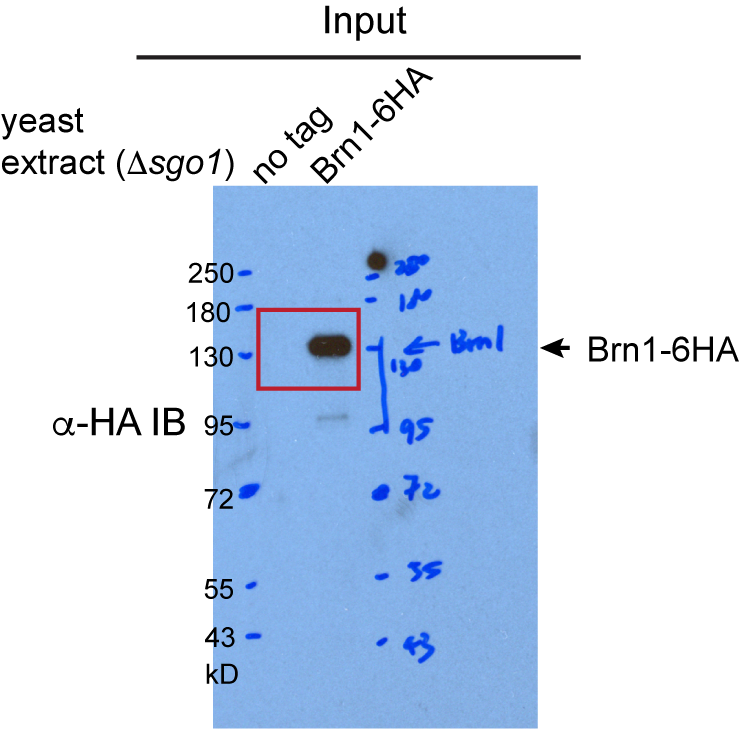

Supplement: Supplementary file 13 — EV Figure Source Data [file 44318_2024_336_MOESM13_ESM.zip › SD EV Figures/FigureEV1/EV1C western INPUT Brn1-HA.tif]

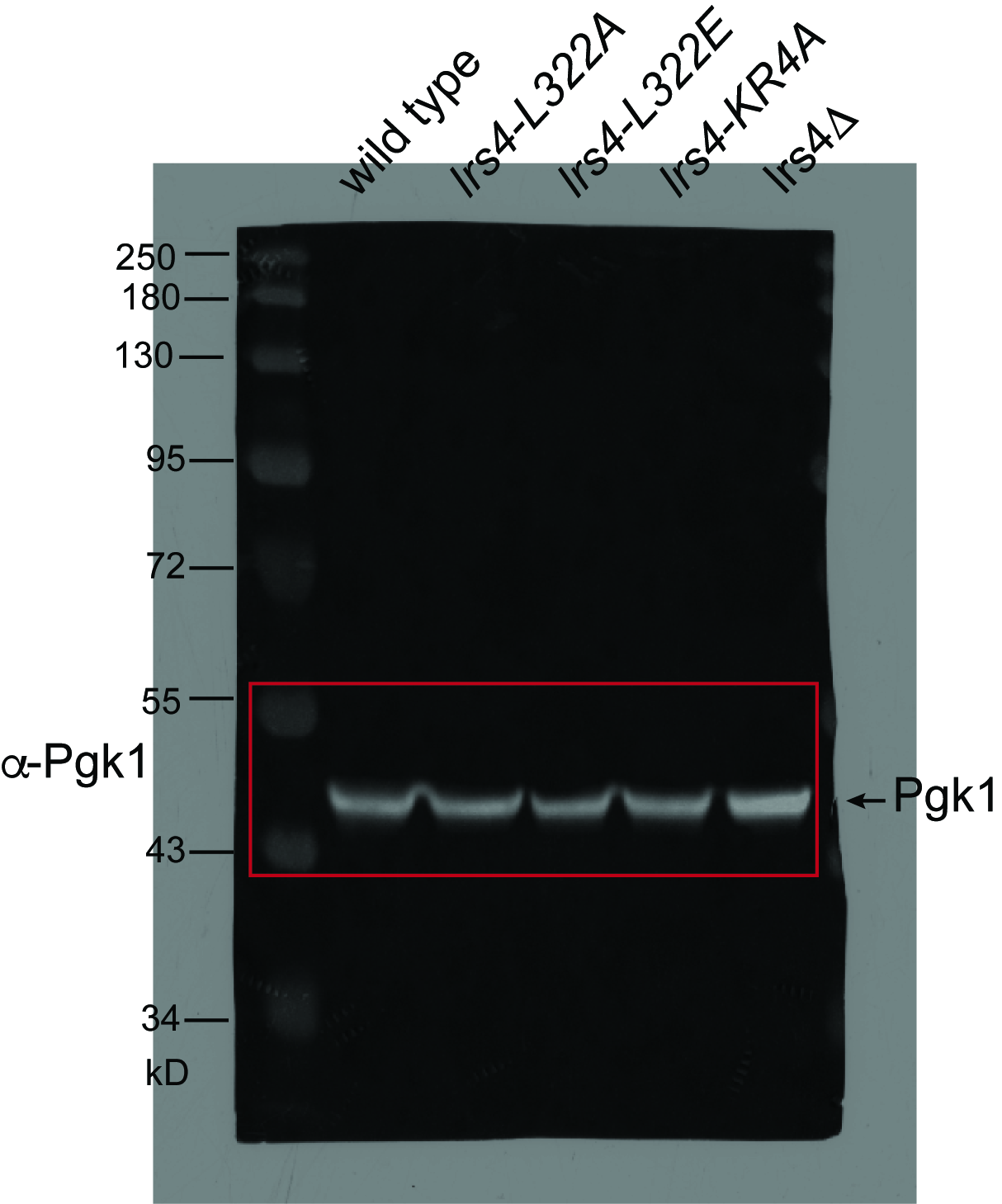

Supplement: Supplementary file 13 — EV Figure Source Data [file 44318_2024_336_MOESM13_ESM.zip › SD EV Figures/FigureEV5/EV5C western PGK1.tif]

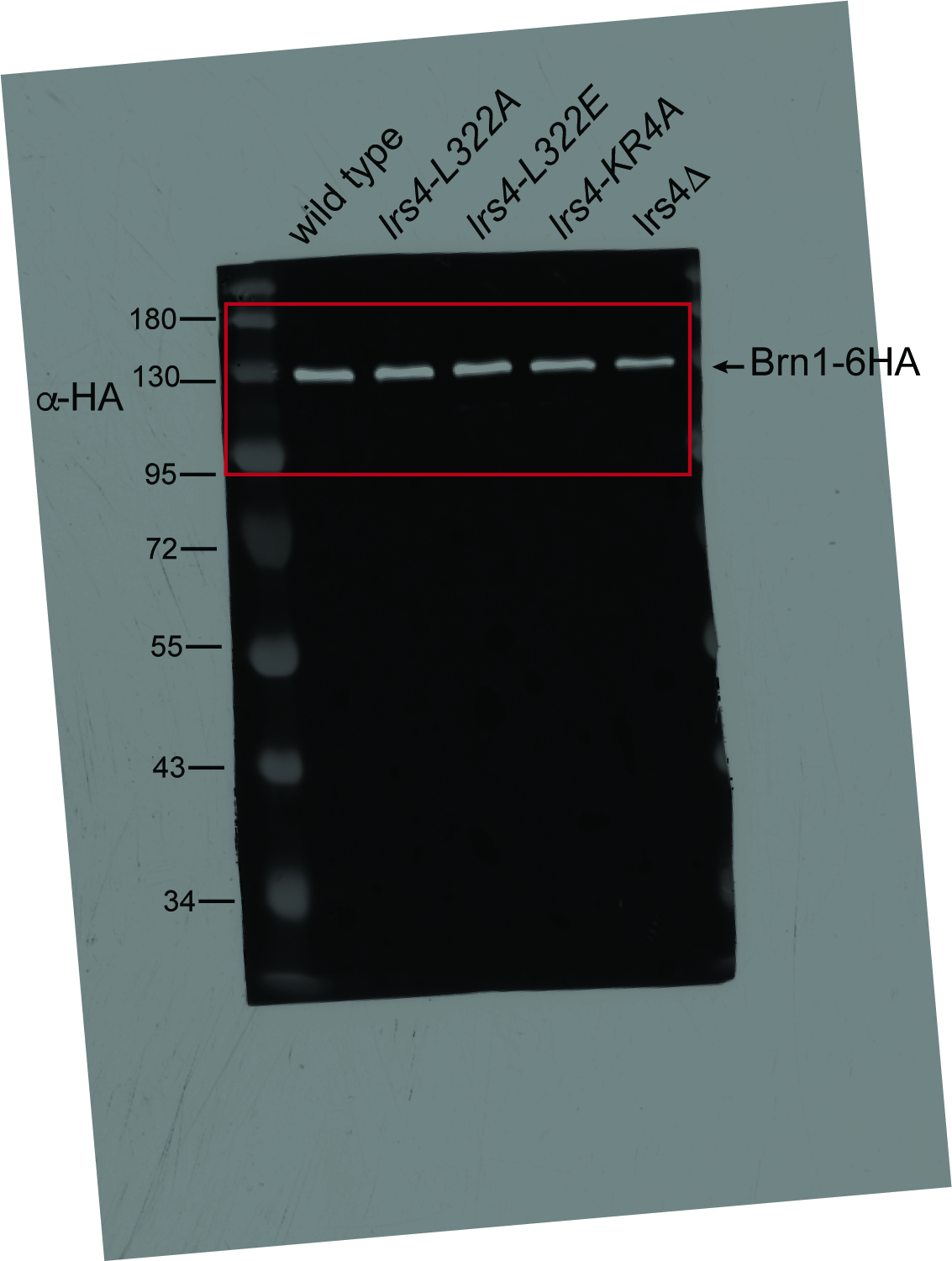

Supplement: Supplementary file 13 — EV Figure Source Data [file 44318_2024_336_MOESM13_ESM.zip › SD EV Figures/FigureEV5/EV5C western Brn1-HA.tif]

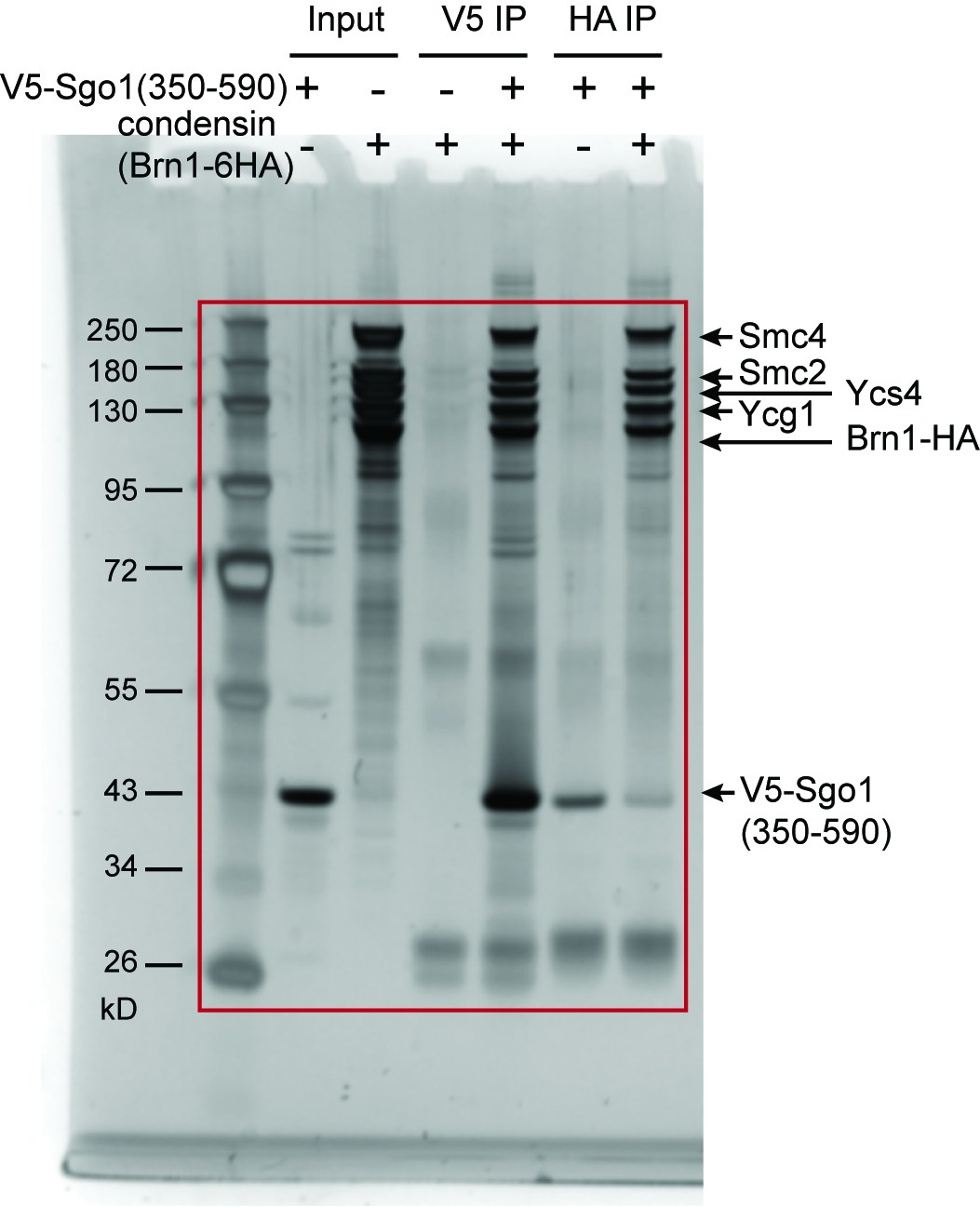

Supplement: Supplementary file 13 — EV Figure Source Data [file 44318_2024_336_MOESM13_ESM.zip › SD EV Figures/FigureEV2/EV2A silver stain.tif]

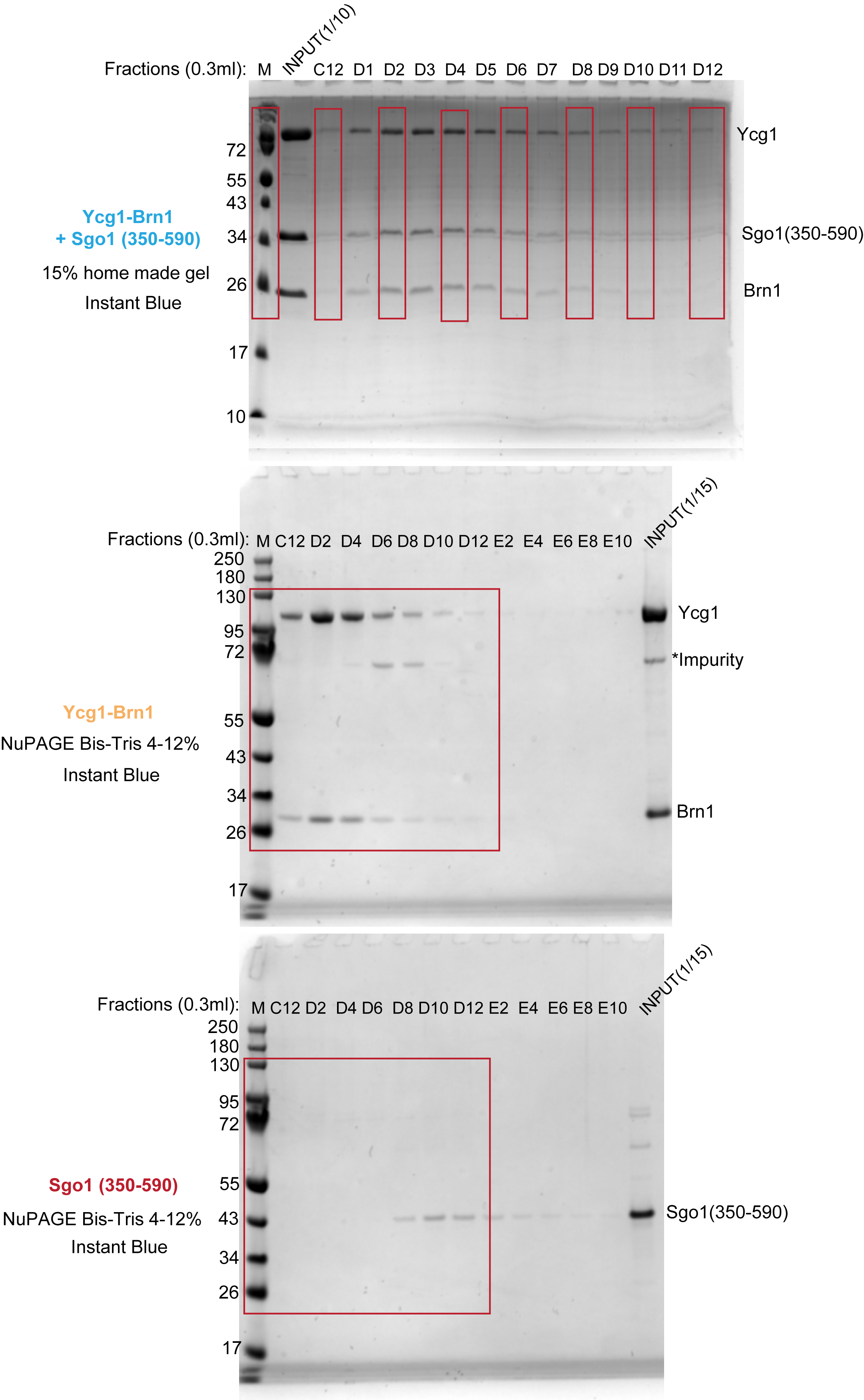

Supplement: Supplementary file 13 — EV Figure Source Data [file 44318_2024_336_MOESM13_ESM.zip › SD EV Figures/FigureEV2/EV2B InstantBlue.tif]

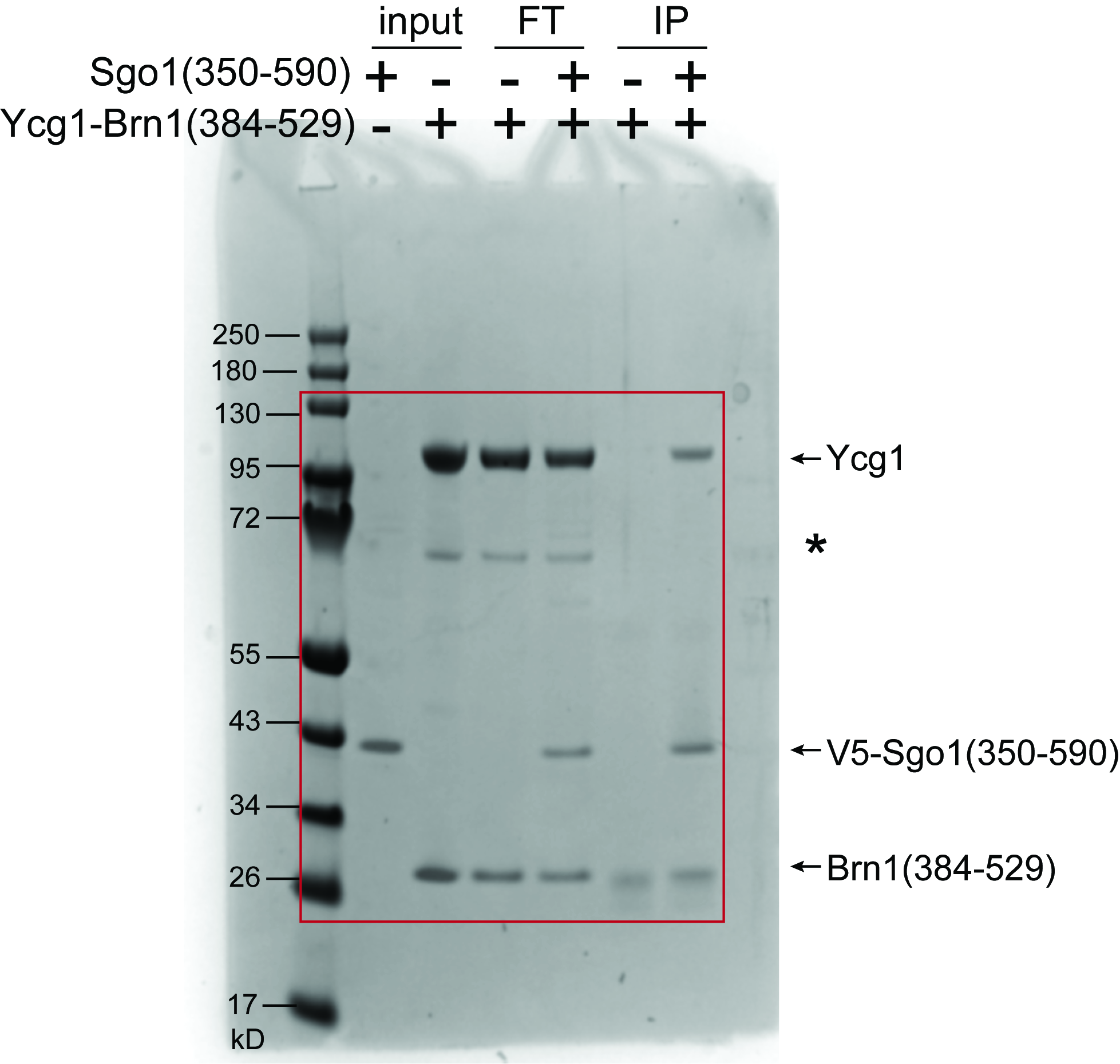

Supplement: Supplementary file 13 — EV Figure Source Data [file 44318_2024_336_MOESM13_ESM.zip › SD EV Figures/FigureEV2/EV2C silver stain.tif]

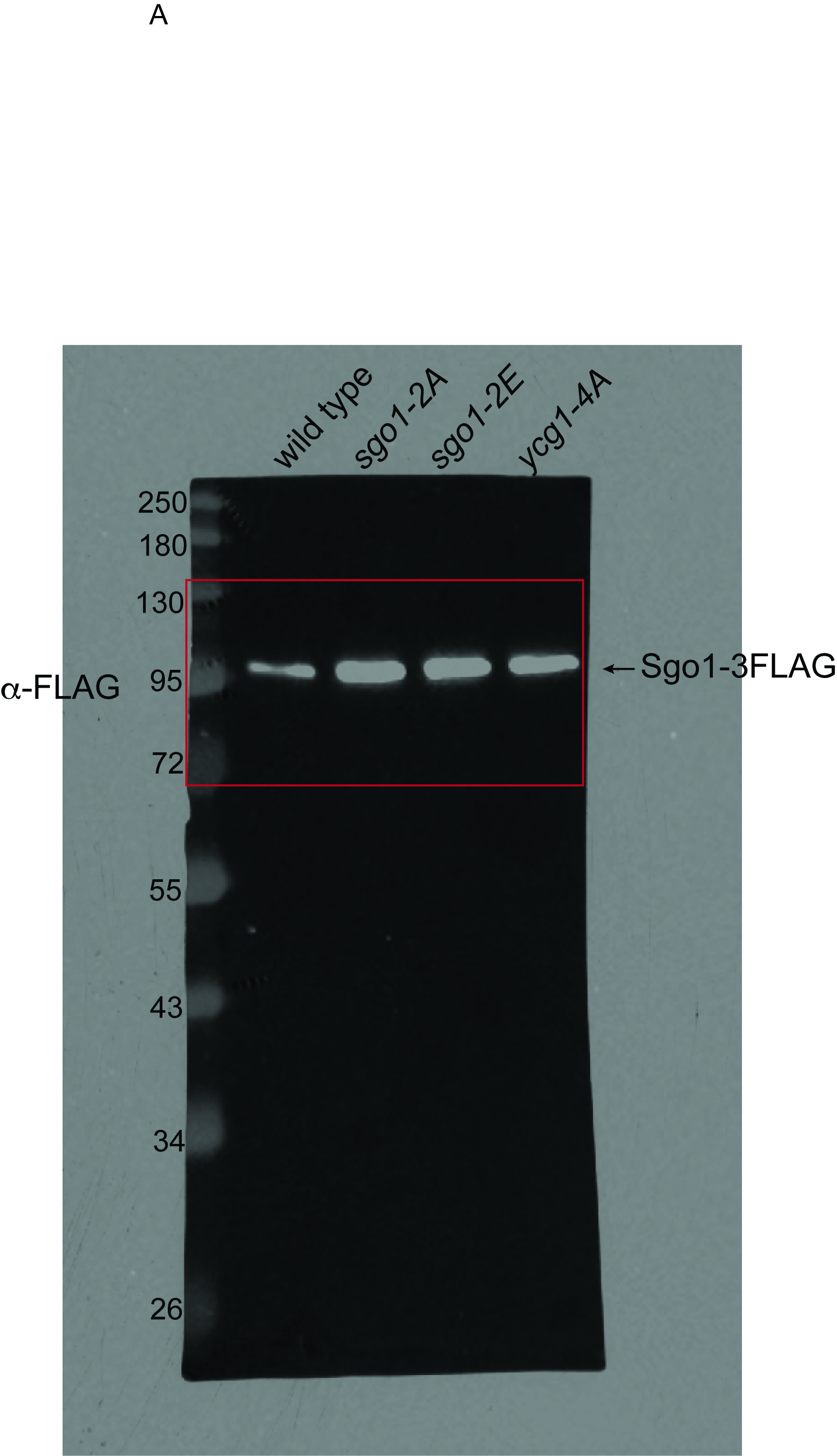

Supplement: Supplementary file 13 — EV Figure Source Data [file 44318_2024_336_MOESM13_ESM.zip › SD EV Figures/FigureEV4/EV4 western Sgo1-FLAG.tif]

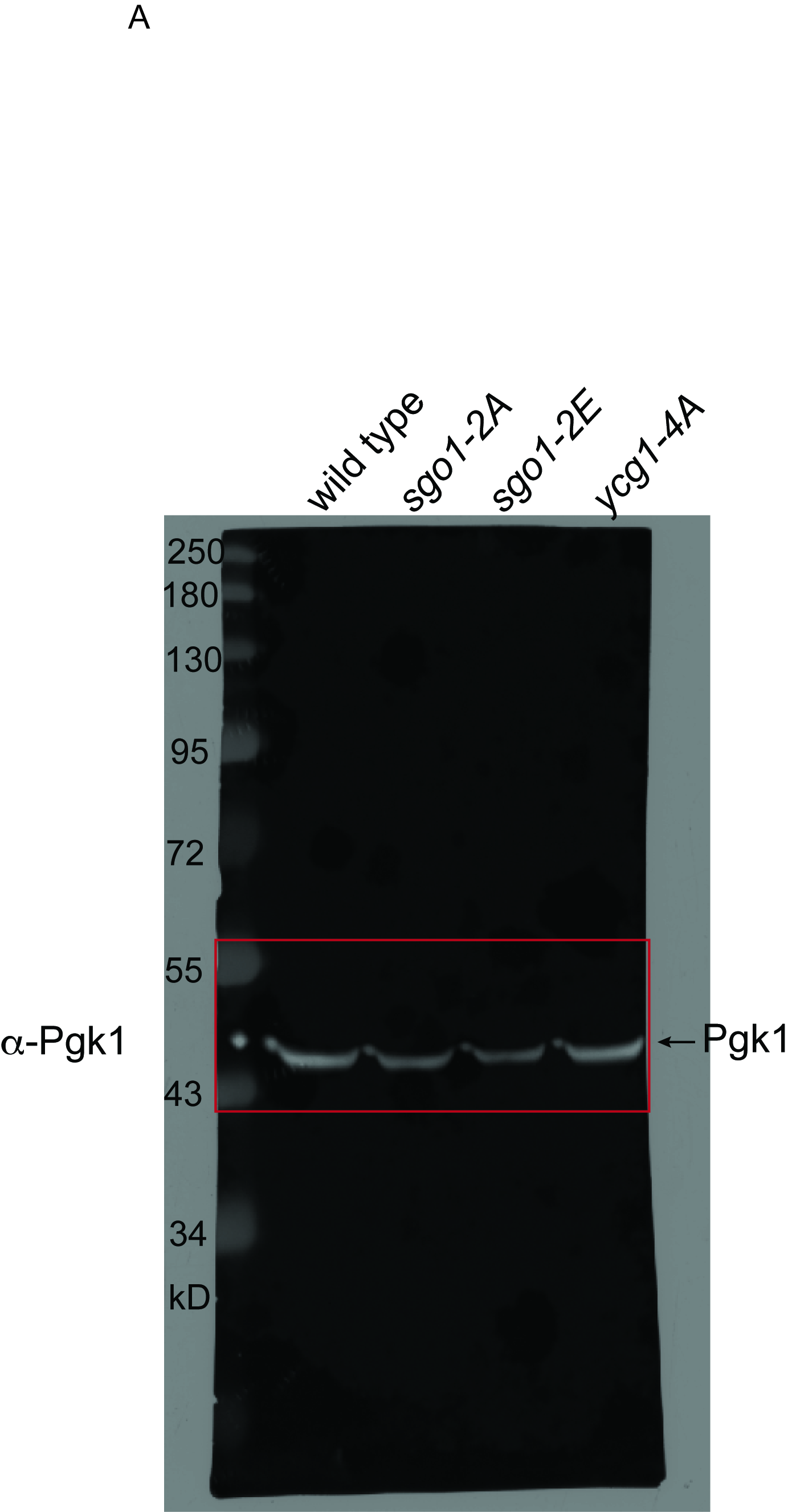

Supplement: Supplementary file 13 — EV Figure Source Data [file 44318_2024_336_MOESM13_ESM.zip › SD EV Figures/FigureEV4/EV4 western PGK.tif]
