# Supplementary material for: Molecular mechanism targeting condensin for chromosome condensation
Source: EMBO J. 2024 Dec 17;44(3):705–35. doi: 10.1038/s44318-024-00336-6 (PMC11791182; doi:10.1038/s44318-024-00336-6)
Supplement: Supplementary file 14 — Expanded View Figures [file 44318_2024_336_MOESM14_ESM.pdf]

Expanded View Figures

Figure EV1. Analysis of Sgo1-condensin complexes.

(**A**) Size exclusion chromatography (SEC) profiles and corresponding silver-stained SDS-PAGE gels and immunoblot using the indicated antibodies for the analysis of full-length Sgo1 (red) and condensin (yellow) complex formation (blue). Note that Sgo1 alone could not readily be detected in this assay as it associates non-specifically with the column in the absence of condensin. The arrowhead indicates the peak of Sgo1-condensin complex. (**B**) Crosslinking mass spectrometry of full-length Sgo1 with condensin data mapped onto reported condensin cryo-EM structure (PDB 6YVU (Lee et al, 2020)) and part Ycg1-Brn1 crystal structure which is a dimer (PDB 5OQQ (Kschonsak et al, 2017)). Self-links are hidden and only crosslinks with score >10.5 are shown. (**C**) Purified GFP-tagged recombinant Sgo1 variants co-immunoprecipitated with condensin (Brn1-6HA) from *sgo1Δ* yeast extract (*sgo1Δ* yeast strains: no tag (AM827), Brn1-6HA (AM8834)). Elutes were analysed by immunoblot with the indicated antibodies. Source data are available online for this figure.

© The Author(s)

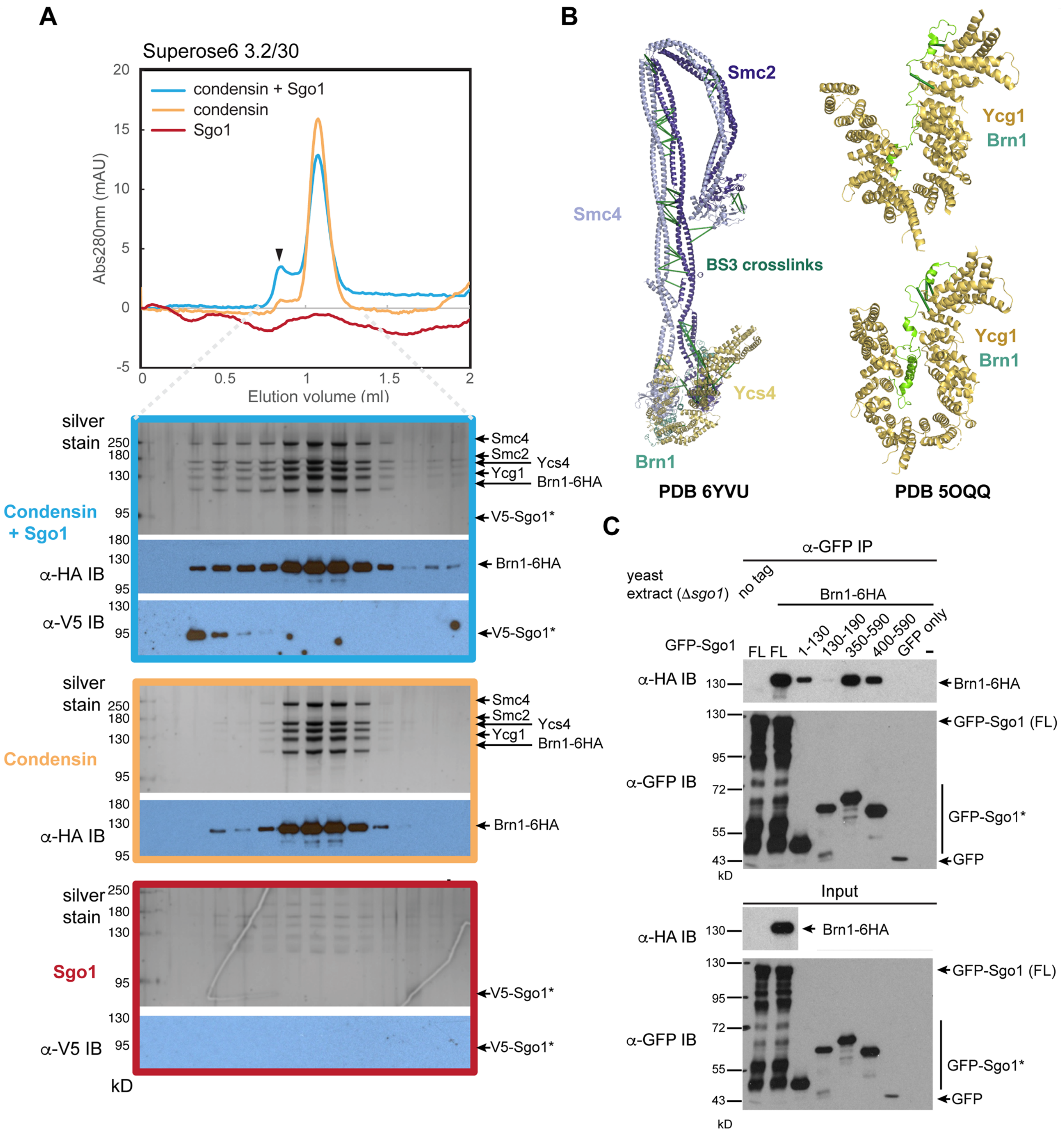
A
Superose6 3.2/30
condensin + Sgo1
condensin
Sgo1
Abs280nm (mAU)
Elution volume (ml)
silver stain
Condensin + Sgo1
α-HA IB
α-V5 IB
Smc4
Smc2
Ycs4
Ycg1
Brn1-6HA
V5-Sgo1*
Condensin
Sgo1
kD
B
Smc2
Smc4
BS3 crosslinks
Ycs4
Brn1
Ycg1
PDB 6YVU
PDB 5OQQ
C
α-GFP IP
yeast extract (Δsgo1)
no tag
Brn1-6HA
GFP-Sgo1
FL FL 1-130 130-190 350-590 400-590 GFP only -
α-HA IB
α-GFP IB
GFP-Sgo1 (FL)
GFP-Sgo1*
GFP
Input
kD

 © The Author(s)

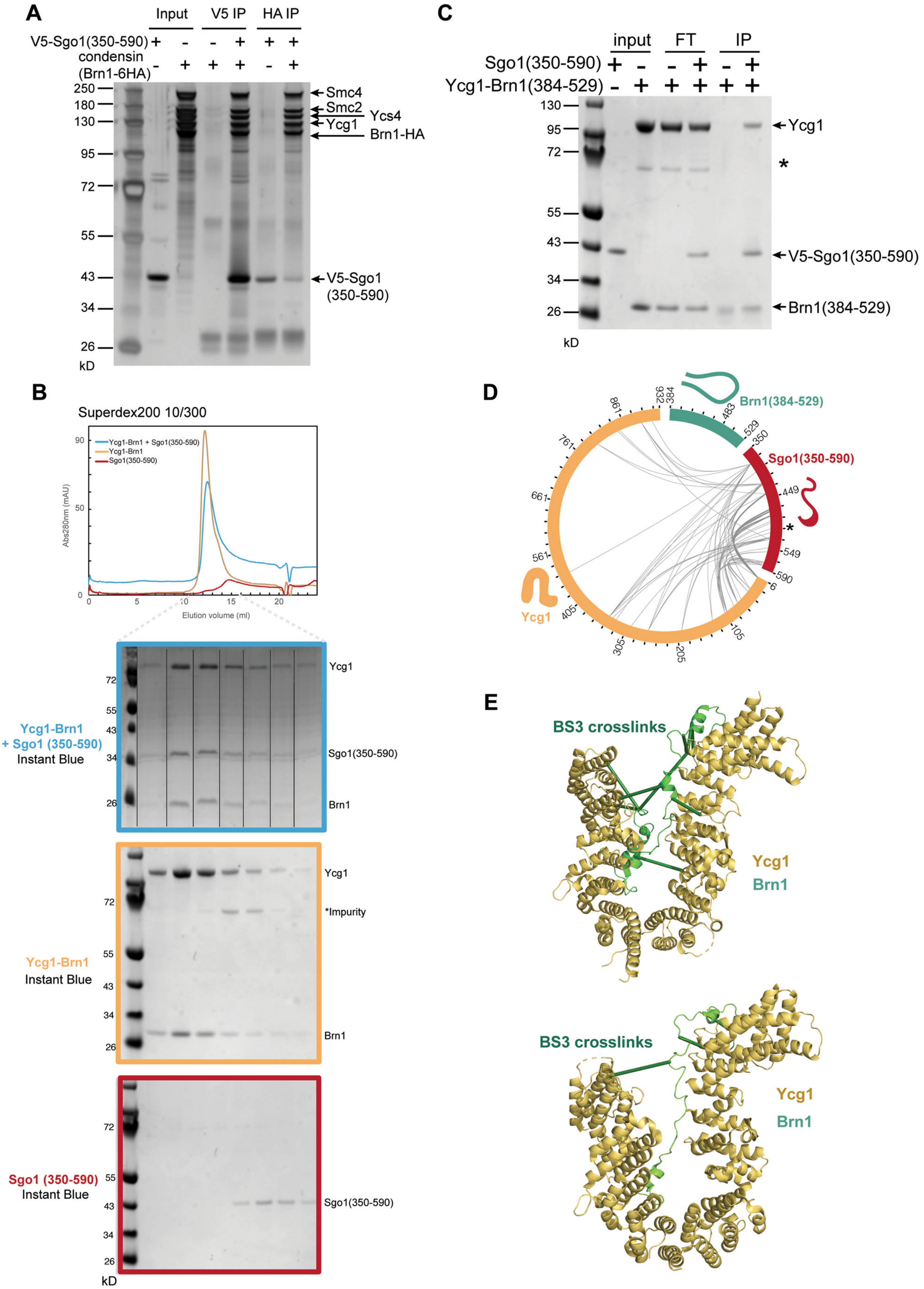
A
Input
V5 IP
HA IP
V5-Sgo1(350-590)
condensin
(Brn1-6HA)
250
180
130
95
72
55
43
34
26
kD
Smc4
Smc2
Ycg1
Ycs4
Brn1-HA
V5-Sgo1
(350-590)
B
Superdex200 10/300
Ycg1-Brn1 + Sgo1(350-590)
Ycg1-Brn1
Sgo1(350-590)
Abs280nm (mAU)
Elution volume (ml)
Ycg1-Brn1
+ Sgo1 (350-590)
Instant Blue
Ycg1
Sgo1(350-590)
Brn1
Ycg1-Brn1
Instant Blue
*Impurity
Sgo1 (350-590)
Instant Blue
C
input
FT
IP
Sgo1(350-590)
Ycg1-Brn1(384-529)
V5-Sgo1(350-590)
Brn1(384-529)
D
Brn1(384-529)
Sgo1(350-590)
Ycg1
E
BS3 crosslinks
Ycg1
Brn1

◀ **Figure EV2. Sgo1 C-terminal region binds directly to Ycg1.**

(**A**) V5 IP: Recombinant C-terminal region of Sgo1 (350–590) co-immunoprecipitated with condensin (Brn1-6HA) using V5-coupled beads. HA IP: Recombinant condensin (Brn1-6HA) immunoprecipitated with V5-Sgo1 (350–590). Note Sgo1(350–590) bound non-specifically to anti-HA beads. Eluates were analyzed by silver-stained SDS-PAGE. (**B**) SEC profiles and corresponding SDS-PAGE for the analysis of interactions (blue) between Sgo1 (350–590) (red) and Ycg1 (6–932, Δ499–555)-Brn1 (384–529) (yellow). Non-relevant lanes have been removed for clarity, as indicated by vertical separation lines. Full gel images are provided in the source data. (**C**) V5 tagged recombinant C-terminal region of Sgo1 (350–590) co-immunoprecipitated with Ycg1 (6–932, Δ499–555)-Brn1 (384–529). Eluates were analyzed by silver-stained SDS-PAGE. Asterisk indicates impurity, FT is flow through. (**D**) BS3 crosslinking mass spectrometry of Sgo1 (350–590) and Ycg1 (6–932, Δ499–555)-Brn1 (384–529). The interactions with Brn1 and self-links are hidden. Crosslinks with score >10.5 are chosen. Asterisk highlights Sgo1 residues (L508, F509) predicted to bind Ycg1. (**E**) Crosslinking mass spectrometry of Sgo1 (350–590) and Ycg1 (6–932, Δ499–555)-Brn1 (384–529) mapped onto Ycg1-short Brn1 crystal structure which is a dimer (PDB 5OQQ (Kschonsak et al, 2017)). Only crosslinks with score >10.5 were shown. Source data are available online for this figure.

 © The Author(s)

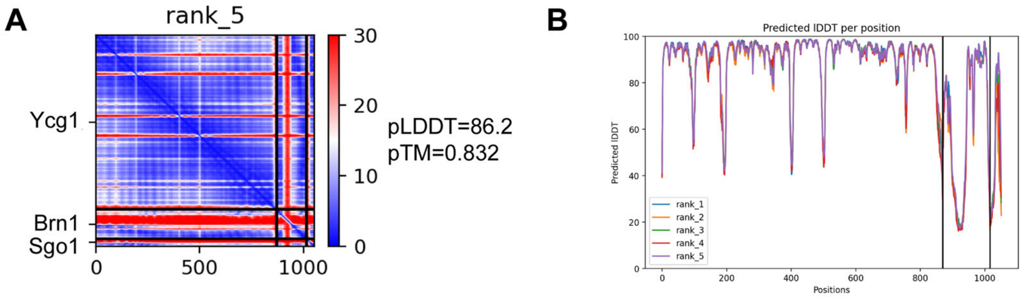

C

pLDDT	Rank1	Rank_2	Rank_3	Rank_4	Rank_5
Sgo1-L508	86.50	79.25	88.06	72.50	88.25
Sgo1-F509	81.56	79.62	86.19	68.94	88.00

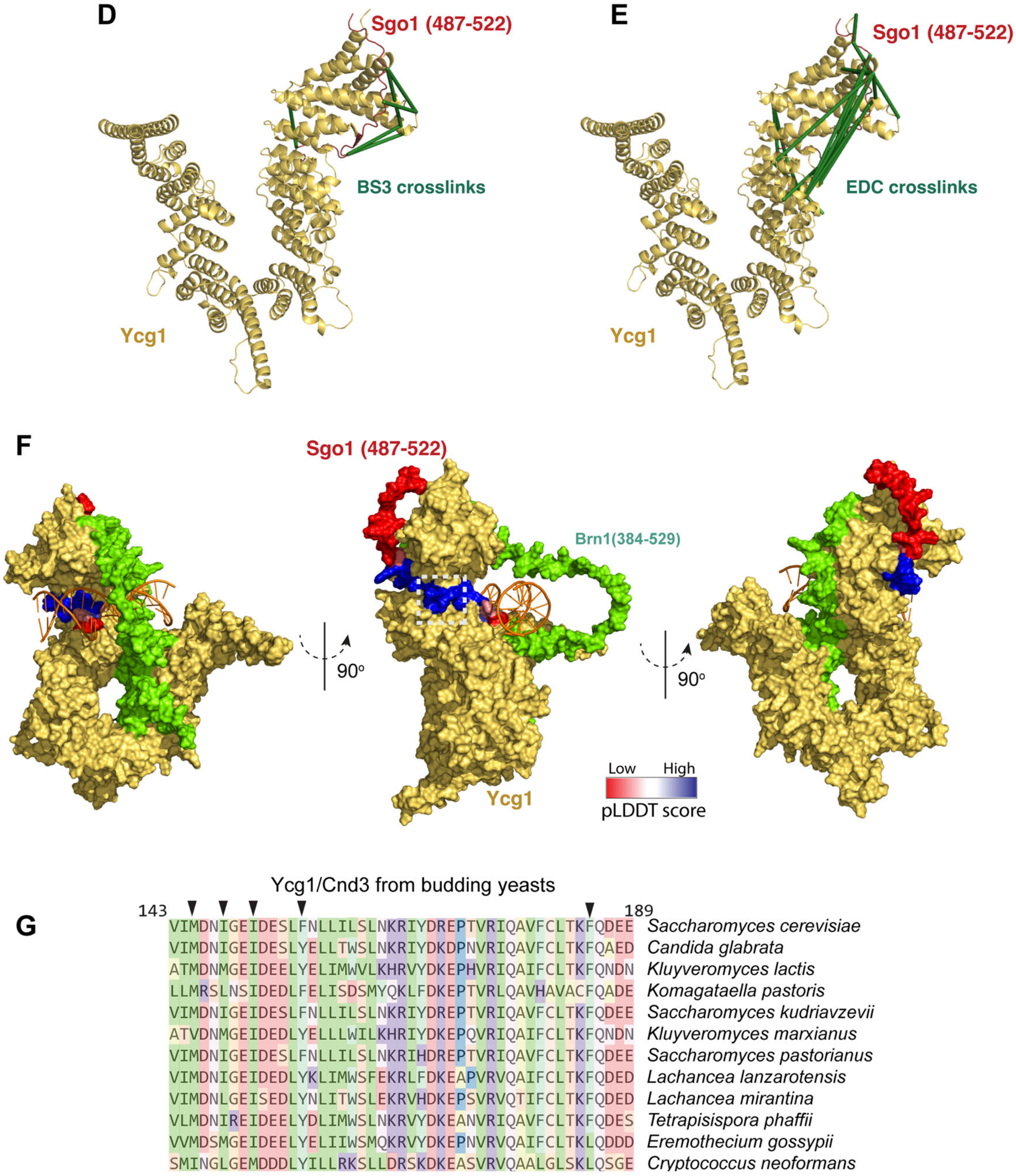

© The Author(s)

Figure EV3. Agreement of the AlphaFold model with CLMS data.

(**A**) Predicted aligned error (PAE) matrices of AlphaFold2 model_Rank 5 obtained from the Sgo1(487–522) with Ycg1 (6–932, Δ499–555)-Brn1 (384–529) prediction. (**B**) pLDDT plot of all 5 AlphaFold2 models. (**C**) Table showing the pLDDT confidence scores of Sgo1 residues L508 and F509 for all five AlphaFold2 models. (**D**, **E**) Crosslinking mass spectrometry data of Sgo1 (350–590) and Ycg1 (6–932, Δ499–555)-Brn1 (384–529) with crosslinker BS3 (**D**) and EDC (**E**) mapped onto the AlphaFold2 model. (**F**) DNA was docked onto the AlphaFold2 model by aligning with the reported crystal structure (PDB 5OQP (Kschonsak et al, 2017)). Sgo1 peptide (487–522) is colored with pLDDT score (blue indicates pLDDT >70, red means pLDDT <50). Sgo1 L508, F509 residues are highlighted in the dashed box. (**G**) Conservation of the Ycg1 binding pocket in related yeast.

 © The Author(s)

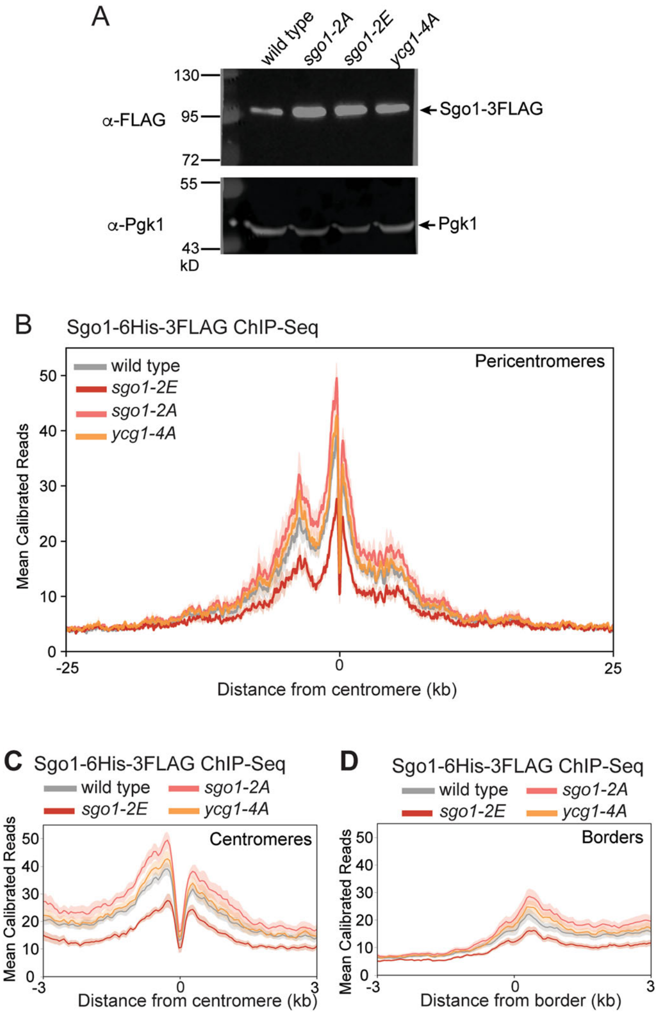

Figure EV4. Sgo1 enrichment along pericentromeres.

(**A**) Anti-FLAG western blotting confirms that Sgo1 is produced at a similar level in all strains. Anti-Pgk1 immunoblot is shown as a loading control. (**B**–**D**) Calibrated Sgo1-6His-3FLAG ChIP-Seq using cells arrested in metaphase by treatment with nocodazole. The pileup of pericentromeric region of all 16 chromosomes (**B**). Zoomed-in pileups of a 6 kb region surrounding 16 centromeres (**C**) or 32 pericentromeric borders (**D**). Strains used in calibrated Sgo1-6His-3FLAG ChIP-Seq: *S. cerevisiae:* wild type (AM32740), *sgo1-2E* (AM33140), *sgo1-2A*(AM33145), *ycg1-4A* (AM33265). *S. pombe* used for calibration: AMsp1863. Source data are available online for this figure.

© The Author(s)

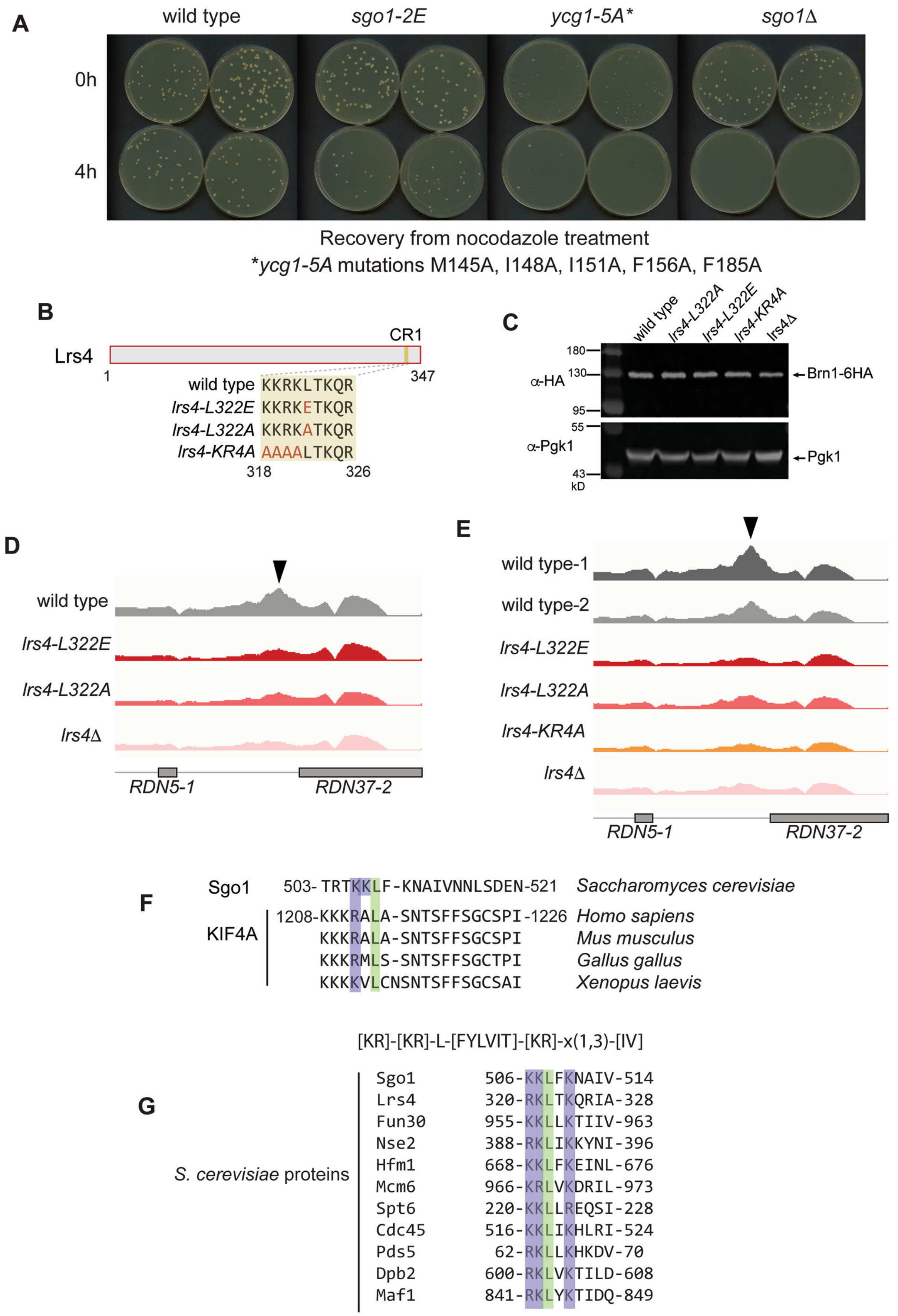
A
wild type
sgo1-2E
ycg1-5A*
sgo1Δ
0h
4h
Recovery from nocodazole treatment
*ycg1-5A mutations M145A, I148A, I151A, F156A, F185A
B
Lrs4
CR1
1
347
wild type KKRKLTKQR
lrs4-L322E KKRKETKQR
lrs4-L322A KKRKATKQR
lrs4-KR4A AAAALTKQR
318
326
C
wild type
lrs4-L322A
lrs4-L322E
lrs4-KR4A
lrs4Δ
180
130
95
55
43
kD
α-HA
α-Pgk1
Brn1-6HA
Pgk1
D
wild type
lrs4-L322E
lrs4-L322A
lrs4Δ
RDN5-1
RDN37-2
E
wild type-1
wild type-2
lrs4-L322E
lrs4-L322A
lrs4-KR4A
lrs4Δ
RDN5-1
RDN37-2
F
Sgo1 503- TRTKKLF-KNAIVNNLSDEN-521 Saccharomyces cerevisiae
KIF4A
1208-KKKRALA-SNTSFFSGCSPI-1226 Homo sapiens
KKKRALA-SNTSFFSGCSPI Mus musculus
KKKRMLS-SNTSFFSGCTPI Gallus gallus
KKKKVLCNSNTSFFSGCSAI Xenopus laevis
G
[KR]-[KR]-L-[FYLVIT]-[KR]-x(1,3)-[IV]
S. cerevisiae proteins
Sgo1 506-KKLFKNAIV-514
Lrs4 320-RKLTKQRIA-328
Fun30 955-KKLLKTIIV-963
Nse2 388-RKLIKKYNI-396
Hfm1 668-KKLFKEINL-676
Mcm6 966-KRLVKDRIL-973
Spt6 220-KKLLREQSI-228
Cdc45 516-KKLIKHLRI-524
Pds5 62-RKLLKHKDV-70
Dpb2 600-RKLVKTILD-608
Maf1 841-RKLYKTIDQ-849

 © The Author(s)

Figure EV5. Identification of CR1-like motifs in other potential Ycg1/CAP-G ligands.

(**A**) Mutation of the Ycg1 binding pocket reduces colony size to a greater extent than mutation of the Sgo1-CR1. Cells were plated onto a rich medium before (0 h) or after the addition of nocodazole (4 h). Strains used: wild type (AM23137), *sgo1-2E* (AM33044), *ycg1-5A* (AM33315), and *sgo1Δ* (AM827). (**B**) Scheme showing the endogenous point mutants generated at the CR1 motif of monopolin protein Lrs4. (**C**) Anti-HA immunoblot showing Brn1 is produced at a similar level in all strains. Anti-Pgk1 immunoblot is shown as a loading control. (**D**, **E**) Calibrated condensin (Brn1-6HA) ChIP-Seq of nocodazole arrested cells at rDNA region. Condensin (Brn1-6HA) enrichment peaks are indicated with arrowheads. *S. pombe* strain AMsp635 was used for calibration. (**D**) FLAG-tagged Lrs4 strains were used: wild type (AM33965), *lrs4-L322E* (AM33967), *lrs4-L322A* (AM33966), *lrs4Δ* (AM9766). (**E**) Untagged Lrs4 strains were used: wild type-1 (AM5708), wild type-2 (AM34390), *lrs4-L322E* (AM34392), *lrs4-L322A* (AM34391), *lrs4-KR4A* (AM34393), and *lrs4Δ* (AM9766). Note two different wild-type mutants were used to confirm that rDNA phenotypes in point mutant strains were not a consequence of their derivation from an *lrs4Δ* parent. Wild type-2 and all mutants were generated from *lrs4Δ* (AM9766) by standard PCR method; while wild type-1 is a standard wild type. (**F**) A CR1 motif is found in KIF4A. (**G**) Potential candidate condensin ligands containing ([KR]-[KR]-L-[FYVIT]-[KR]-x(1,3)-[IV]) in *S. cerevisiae*. Source data are available online for this figure.

© The Author(s)
